# A Highly Sensitive Water-Soluble Donor–Acceptor Dye for Early-Stage Amyloid Aggregation Kinetics

**DOI:** 10.1021/acs.jpcb.5c07705

**Published:** 2026-03-10

**Authors:** Giorgio Scattolini, Carlos Enrique Torres-Méndez, Dylan Valli, Mikołaj Ignacy Kuska, Nidhi Kaul, Leif Hammarström, Haining Tian, Michał Maj

**Affiliations:** Department of Chemistry − Ångström Laboratory, 8097 Uppsala University, Box 523, 751 20 Uppsala, Sweden

## Abstract

Protein aggregation into amyloid fibrils underlies numerous human diseases, yet the most widely used fluorescent probe, Thioflavin T (ThT), offers an incomplete picture of the process and fails to detect certain fibril structures. Here, we introduce and characterize the photophysical properties of DANIR-2b­(2OH), a water-soluble push–pull dye that overcomes these limitations. It successfully binds early prefibrillar aggregates and small fibrils of the human Islet Amyloid Polypeptide that elude detection by ThT, which we confirm by time-resolved cryo-electron microscopy of aliquots taken during the kinetic assays. We further demonstrate that DANIR-2b­(2OH) can also track the aggregation of other amyloid proteins, such as insulin and Aβ_1–42_. The protein-dye interaction was characterized via steady-state and time-resolved fluorescent spectroscopy. DANIR-2b­(2OH) features environment-sensitive emission, high photostability, and a straightforward synthesis. Critically, it provides a substantially lower noise level in standard plate-reader assays, allowing the tracking of aggregation processes that are not visible in standard ThT measurements. This establishes DANIR-2b­(2OH) as a highly sensitive and broadly applicable probe for real-time amyloid aggregation measurements and imaging.

## Graphical abstract

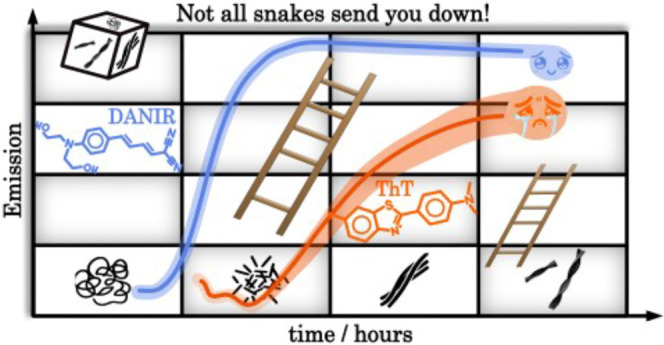

## Introduction

The misfolding and aggregation of proteins into amyloid fibrils is linked to the development of many human diseases.^1–3^ The ability to monitor the kinetics of the aggregation process in real-time is key to understanding the underlying mechanisms. It is also the most quantifiable method for determining amyloidogenic propensity of proteins, which differs by the length of the lag phase period associated with the initial nucleation steps.^4,5^ Studying changes in aggregation kinetics also provides a straightforward way of testing inhibitors or small molecules capable of disaggregating fibrillar plaques.^6–8^ Such compounds hold great promise as potential drug candidates for neurodegenerative disorders.

The kinetics of amyloid aggregation are typically measured using environment-sensitive fluorescent probes.^9–11^ Less commonly, scattering techniques can be used either independently or alongside fluorescence assays, but these are less frequently employed due to the need for more demanding instrumentation.^12^ Fluorescent measurements, on the other hand, are conveniently implemented in plate readers, allowing for the simultaneous analysis of hundreds of samples. In the search for inhibitors of heparin-induced tau fibrillation, over 51,000 compounds have been screened with such a fluorescence assay.^13^ Similar high-throughput strategies have been used for years to screen for other amyloid proteins such as α-synuclein,^14,15^ Amyloid β,^16^ or prion protein.^17^

A fluorescent dye used in amyloid aggregation studies should exhibit a high affinity and specificity for binding to amyloids. Second, its photophysical properties should change in the presence of amyloids, distinguishing bound dye from free dye in solution. Lastly, the dye must act as a passive observer of the aggregation process without interfering with the kinetics. By far, the most used dye to study the formation of amyloid fibrils is thioflavin T (ThT), a molecular rotor whose fluorescence quantum yield (Φ_f_) is enhanced by several orders of magnitude when bound to β-sheets (Figure 1).^18^ Structurally, ThT comprises a benzothiazole ring connected via a monomethine bridge to a dimethylaminobenzene moiety. In aqueous solution, the two aromatic rings rotate freely around the central C–C bond, and the resulting intramolecular rotation opens a nonradiative, twisted-intramolecular-charge-transfer (TICT) pathway that makes free ThT only weakly fluorescent.^19^ Upon binding to amyloid fibrils, ThT intercalates into linear surface grooves that run parallel to the fibril axis in the cross-β architecture.^18,20^ In this restricted environment, the rotation along the C–C bond is hindered, preventing the decay of the system to the TICT state, leading to an increase in Φ_f_ as the aggregation process proceeds.^21^

**Figure 1:**
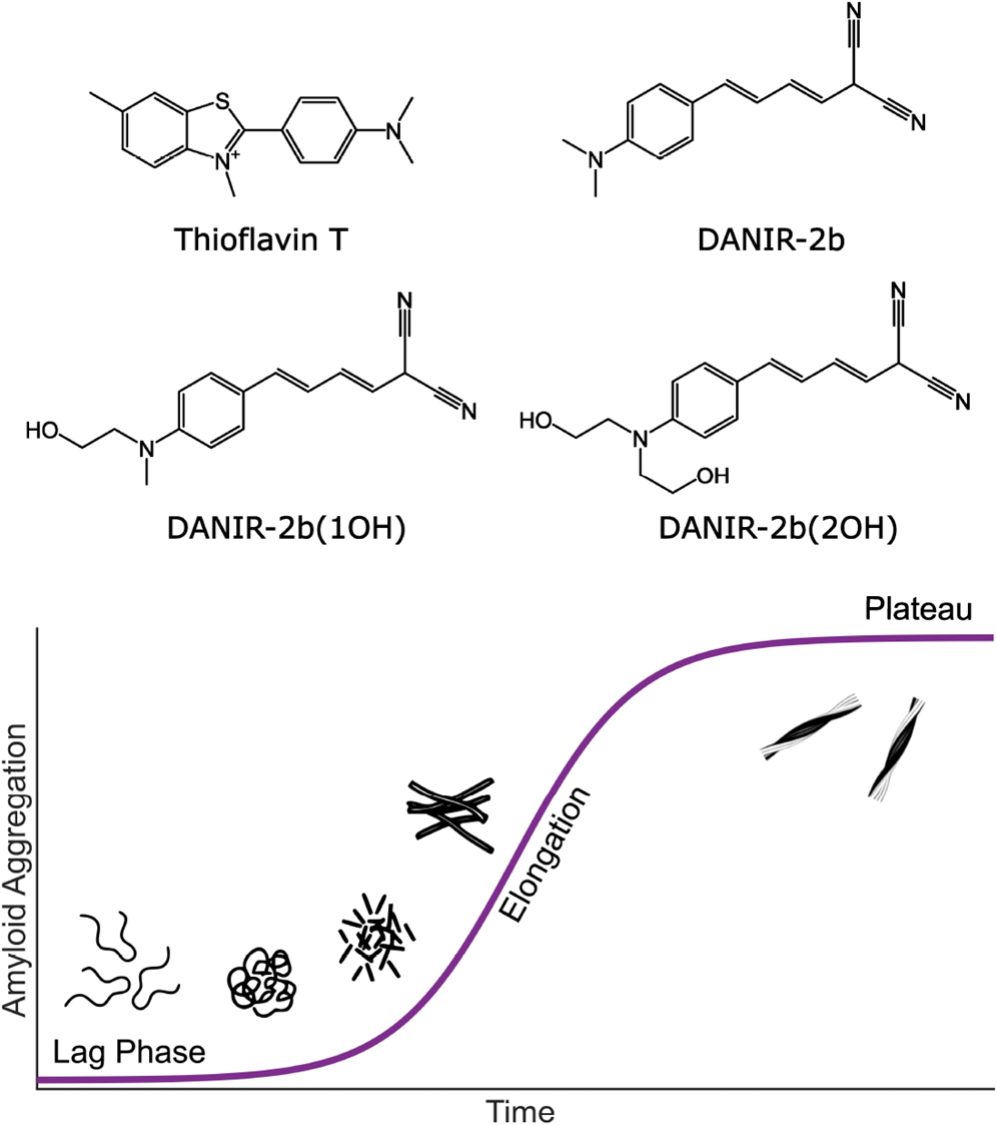
(Top) Chemical structures of thioflavin T, DANIR-2b (parent compound), DANIR-2b­(1OH), and DANIR-2b­(2OH). (Bottom) Schematic representation of the characteristic sigmoidal aggregation curve, indicating the lag, elongation, and plateau phases referenced throughout this study.

Despite its widespread use, ThT exhibits several practical limitations in amyloid studies. A noteworthy drawback is its high variability in binding constants and fluorescence intensities across different fibril structures.^22–25^ The Islet Amyloid Polypeptide (IAPP) from Pufferfish (*Takifugu rubripes*) forms fibrils that produce no ThT signal, leading to false negatives in standard assays.^23^ Similarly, the Japanese mutant (ΔE22) of Amyloid β_1–39_ binds very little ThT, which is attributed to its highly twisted helical structure, resulting in limited binding sites.^24^ Our recent cryo-electron microscopy (cryo-EM) study has also demonstrated that the ThT exhibits higher affinity to nonhelical polymorphs of the human Islet Amyloid Polypeptide (hIAPP) and that changes in solution composition have a dramatic effect on the measured signals, with certain conditions completely arresting binding.^25^ In addition, although ThT remains the standard readout for in vitro aggregation kinetics, its performance in microscopy applications is variable. In fixed tissue, many laboratories use Thioflavin-S (ThS) for robust plaque labeling, whereas ThT is used less frequently and can show additional cellular staining that complicates interpretation.^26^

Consequently, there has been an ongoing effort to develop alternative dyes for protein aggregation for both in vitro and imaging studies.^9,10,27^ The research focuses mostly on three directions: improving the photochemical properties of dyes, distinguishing structural polymorphs, and detecting oligomers. Examples of improving the photochemical properties of dyes include Thioflavin-X, which exhibits greater brightness and affinity for amyloids than ThT.^28^ Furthermore, it allows for single-molecule microscopic localization and binds to oligomers. Another example is YAT 2150, which binds to fibrils better than ThT both in vitro and in vivo.^29^ However, its use to track aggregation kinetics is limited as it has also been reported as an inhibitor of protein aggregation.^30,31^

The differentiation of amyloid polymorphs of the same protein by different dyes represents another goal of the design of aggregation monitoring dyes. Examples that address this problem are polythiophens, which are able to report different types of polymorphs based on the conformational changes induced by the binding event.^32^ Another key challenge in dye development is the detection of oligomers, given their transient nature and cytotoxicity.^33^ For example, the dyes taBODIPY and AN-SP are capable of binding prefibrillar structures or oligomers.^34^ However, they do not bind to α-synuclein fibrils; they present limited water solubility and poor signal quality.

A different strategy for the detection of oligomeric and prefibrillar structures is based on monitoring energy transfer between different chromophores and how this process is affected by the association of the dyes to the fibrils during the aggregation.^35,36^ This approach has been shown to be effective, but its reliance on multiple components introduces synthetic complexity and higher chances of interference with the aggregation process.

Another interesting group of chromophores is push–pull dyes, characterized by high sensitivity to dielectric environment and binding geometry.^11^ One such family of push–pull molecules, which forms the basis for this study, is the DANIR dyes, which feature a dimethylamino electron donor and a malononitrile acceptor.^37–39^ The original parent compound is DANIR-2b (Figure 1). This dye and an elongated analogue (DANIR-2c) were introduced by Cui et al. as two-photon imaging agents for Aβ fibrils.^37^ Watanabe et al. later demonstrated binding of these DANIR dyes and their monohydroxy analogues (including DANIR-2b­(1OH), Figure 1) to hIAPP fibrils.^38^ Despite these promising characteristics, the proposed monohydroxylated analogue was combined with ∼5% v/v dimethyl sulfoxide (DMSO) for complete solubilization in water, which is undesirable in kinetic studies because cosolvents such as DMSO can alter peptide solvation and modulate fibril formation.^40,41^ Recently, more sophisticated hydroxylated analogues of naphthalene-based DANIR dyes were introduced as in vivo staining agents for Aβ fibrils, but their applicability to studying aggregation kinetics was not determined.^42^

Here, we introduce DANIR-2b­(2OH), a derivative designed as a nonperturbative probe of protein aggregation that retains the desirable photophysics of the DANIR series while exhibiting substantially improved solubility in aqueous media. We characterize the stability and photophysical properties of DANIR-2b­(2OH) and demonstrate that it reports amyloid binding with high sensitivity at submicromolar dye concentrations and improved signal stability. Importantly, DANIR-2b­(2OH) allows us to follow the aggregation kinetics of fibrils that yield little to no signal when studied with ThT, including pufferfish IAPP. We foresee DANIR-2b­(2OH) to become one of the mainstream assays in amyloid studies due to its straightforward synthetic route and broad utility in both kinetic assays and imaging.

## Experimental Section

### Ab Initio Calculations

All quantum chemical calculations were performed using the Gaussian 16, Revision C.01 program package.^43^ Geometries for both the ground state (S_0_) and the first singlet excited state (S_1_) were fully optimized by using density functional theory (DFT) and its time-dependent extension (TD-DFT), respectively. All optimizations were performed using the CAM-B3LYP range-separated functional,^44^ which is well-suited for describing charge-transfer states, combined with the 6-311++G­(d,p)^45^ basis set. Calculations were carried out both in vacuum (gas phase) and in implicit solvent. Solvatochromic effects of water were evaluated using the integral equation formalism polarizable continuum model (IEF-PCM).^46^ Vertical absorption energies (λ_abs_) were computed via single-point TD-DFT calculations (requesting 6 states) at the optimized S_0_ geometries. To predict the emission spectra (λ_em_), the first excited state was optimized, and the subsequent vertical de-excitation energy was calculated from the relaxed S_1_ geometry. Stokes shifts were determined as the difference between the vertical absorption and emission energies.

### UV–Vis Absorption and Emission Spectroscopy

UV–vis absorption measurements were performed with a Cary 60 spectrophotometer (Agilent) with a 1 cm quartz cuvette.

Fluorescence measurements were performed using a Fluorolog-3 spectrofluorimeter (Horiba). The samples of DANIRs in different solvents were loaded in a 1 cm quartz cuvette, and their emission was recorded in right-angle geometry. The measurements were conducted by exciting the samples at 450 nm, with the excitation slit set at 4 nm, the emission slit set at 3 nm, and an integration time of 0.1 s.

### Quantum Yield Determination

[Ru­(bpy)_3_]^2+^ in water (Φ_f_ = 0.04)^47^ was used as a standard to determine the quantum yield of emission of both dyes. Absorbances of sample and standard were matched at the excitation wavelength, and emission was measured under identical conditions (λ_ex_ = 470 nm, excitation and emission slits = 3 nm, and integration time= 0.1 s). Care was taken to keep sample absorbances <0.1 to avoid inner filter effects.

### Time-Correlated Single Photon Counting

Time-correlated single photon counting (TCSPC) measurements were performed with a FS5 spectrofluorimeter (Edinburgh Instruments), in a time window of 50 ns, using an EPL475 pulsed diode laser source of 475 nm as an excitation source (full width at half-maximum = 0.9 ns) (Edinburgh Instruments). The samples were loaded in a 10 μm quartz cuvette, and the emission was recorded in front-face geometry.

The instrument response function was determined by setting the emission monochromator at the same wavelength as the laser diode and by measuring the light scattered by a sample containing only water, loaded in the same cuvette used for the protein sample.

### Peptide Synthesis and Purification

Amyloid β_1–42_ was purchased from Bachem (Product number: 4014447), and human recombinant insulin was purchased from Sigma (Product number: 91077C). hIAPP was synthesized on an Initiator Alstra (Biotage) using standard Fmoc-based solid-phase methods. TentaGel R RAM resin was used to yield peptides with an amidated C-terminus, and incorporation of pseudoproline dipeptides followed established protocols.^48^ The product was cleaved from the resin using a mixture of TFA:TIS:H_2_O (95:2.5:2.5), dissolved in H_2_O, and lyophilized overnight. The disulfide bridge between C2 and C7 was formed by dissolving the peptides in a 60:40 mixture of DMSO and acetic acid for 24 h. Purification was performed using reverse-phase high-performance liquid chromatography (ISERA C18 preparative column). The purified peptides were lyophilized overnight, then resuspended in 100% hexafluoroisopropanol (HFIP) and sonicated to monomerize the peptides before being aliquoted. The aliquots were lyophilized to remove HFIP and stored at – 20 °C until use. The mass of the peptides was confirmed using liquid chromatography–mass spectrometry.

### Aggregation Kinetics

Aggregation kinetics were monitored by measuring the increase in the fluorescence intensity of ThT or DANIR-2b­(2OH) on a Victor X4 microplate reader (PerkinElmer). All samples were measured in triplicate in a 96-well black plate covered with a black TopSeal-A membrane to prevent evaporation. ThT fluorescence was measured by using an excitation wavelength of 450 nm and an emission wavelength of 490 nm. DANIR-2b­(2OH) fluorescence was measured by using an excitation wavelength of 490 nm and an emission wavelength of 590 nm. For insulin experiments, the dry peptide was initially solubilized in 10 mM HCl, immediately neutralized, and further diluted in aggregation buffer with 20 mM dithiothreitol to a final peptide concentration of 50 μM.

Full spectral kinetics were measured using a FS5 spectrofluorimeter (Edinburgh Instruments) coupled to a home-built sample stage via a SC-50 optical fiber sample module (Edinburgh Instruments); 100 μL of the sample was loaded into a cell made up of two CaF_2_ windows and a 2 mm Teflon spacer that was kept horizontal during the measurement. The sample was excited at 445 nm using a continuous wave laser, and the emitted light was collected with a fiber optic and directed toward the detector.

### Atomic Force Microscopy

Samples for atomic force microscopy were taken directly from the well plate used for aggregation kinetics. A 20 μL aliquot of each sample was deposited onto a mica substrate and incubated for 15 min. The substrate was then rinsed three times with 50 μL of deionized water, and excess water was removed using a wiper (Kimwipe) after each wash. Images were acquired using a Dimension ICON4-SYS atomic force microscope (Bruker) and processed using NanoScope Analysis 1.9 software.

### Cryo-Electron Microscopy

The samples for cryogenic electron microscopy were taken directly from the wells used for aggregation kinetics measurements. QuantiFoil 3.5/1 grids were glow-discharged for 60 s at 20 mA, and 4 μL of sample was applied. After a waiting time of 30 s, the grid was blotted for 0.5 s and plunge-frozen in liquid ethane with a Vitrobot Mark IV (Thermo Scientific). Imaging was performed using a 200 kV Glacios cryo-TEM equipped with a Falcon4i detector (Thermo Scientific). All micrographs were taken at a magnification of 79,000× and a defocus of −2 μm with a total electron dose of 45 e/Å^2^.

### Two-Photon Fluorescence Imaging

INS1-E cells were seeded on poly-l-lysine-coated glass coverslips and cultured in RPMI 1640 medium supplemented with 10% fetal bovine serum at 37 °C in a humidified incubator with 5% CO_2_. The coverslips were washed twice with phosphate-buffered saline and fixed with cold methanol at −20 °C for 20 min. Cells were then washed with PBS, incubated with DANIR-2b­(2OH) at 100 μM for 30 min in the dark, and washed with PBS before mounting. Two-photon fluorescence imaging was performed on a LEICA SP8 DIVE microscope using a 980 nm excitation wavelength and collecting emission between 541 and 611 nm.

## Results

### Molecular Design and Synthesis

Designing the dye, we aimed at improving DANIR’s aqueous solubility without perturbing its donor–acceptor electronic structure and its photophysical properties. Rather than sulfonation, which adds synthetic complexity, we introduced hydroxyl groups on both alkyl substituents of the dimethylamino donor to yield DANIR-2b­(2OH), guided by prior evidence that a single hydroxyl does not impair amyloid binding.^38^ TD-DFT calculations in a polarizable continuum model (PCM) indicate that DANIR-2b­(2OH) maintains a bright S_0_ → S_1_ charge-transfer excitation dominated by a HOMO→LUMO (HOMO: Highest Occupied Molecular Orbital, LUMO: Lowest Unoccupied Molecular Orbital) transition and a near-planar backbone. Thus, the hydroxyls primarily enhance solubility with a minimal impact on the orbital character. Following the excited state calculations, we predict the emission spectra by optimizing gradients in the lowest-lying excited state and calculating the Stokes shifts. The calculated excitation energies exhibit a consistent blue-shift of ≈ 70 nm compared to experimental values, which is expected for this level of theory. The calculated Stokes shifts are in excellent agreement with the experiments. This confirms that the additional hydroxyethyl group does not significantly alter the photophysical properties of the dye. Summary data appear in Table 1, and molecular orbitals are provided in Figure S1 of the Supporting Information.

**Table 1:** Experimental and Calculated Absorption and Emission Maxima (nm) for DANIR-2b­(1OH) and DANIR-2b­(2OH) in Water (λ_abs,H_2_O_, λ_em,H_2_O_) and in the Gas Phase (λ_abs,vac_, λ_em,vac_)

	**experiments**		**calculations**			
**compound**	**λ_abs,H2O_**	**λ_em,H2O_**	**λ_abs,H2O_**	**λ_em,H2O_**	**λ_abs,vac_**	**λ_em,vac_**
DANIR-2b(1OH)	490	592	418	520	382	410
DANIR-2b(2OH)	486	592	413	517	381	410

The synthetic route to DANIR-2b­(2OH) (and DANIR-2b­(1OH)) is shown in Figure 2. Both dyes were obtained in four steps from the corresponding 4-(dialkylamino)­benzaldehydes. First, the hydroxyl groups on the amino substituent were protected as tetrahydropyranyl ethers using 3,4-dihydro-2H-pyran and catalytic pyridinium *p*-toluenesulfonate in dichloromethane at 40 °C. Then, extension of the π-bridge was achieved by the Wittig reaction of the aryl aldehyde with (1,3-dioxan-2-ylmethyl)­triphenylphosphonium bromide (NaH, 18-crown-6, tetrahydrofuran (THF), 20 °C), forming the corresponding styryl aldehydes, which underwent subsequent Knoevenagel condensation with malononitrile in ethanol (EtOH) at 20 °C to deliver the dicyanovinyl acceptor. Finally, global acidic deprotection (1 M HCl, EtOH, 20 °C) produced the free hydroxyl groups. Performing the deprotection of the hydroxyl groups as the last step of the synthesis and performing the Knoevenagel condensation under catalyst-free conditions allowed us to increase the yield nearly 8-fold compared to the previous protocols.^38^ The detailed synthetic procedure and the characterization of the final products and of the reaction intermediates by mass spectrometry and ^1^H and ^13^C nuclear magnetic resonance spectroscopy are provided in the Supporting Information (Figures S2–S14).

**Figure 2:**
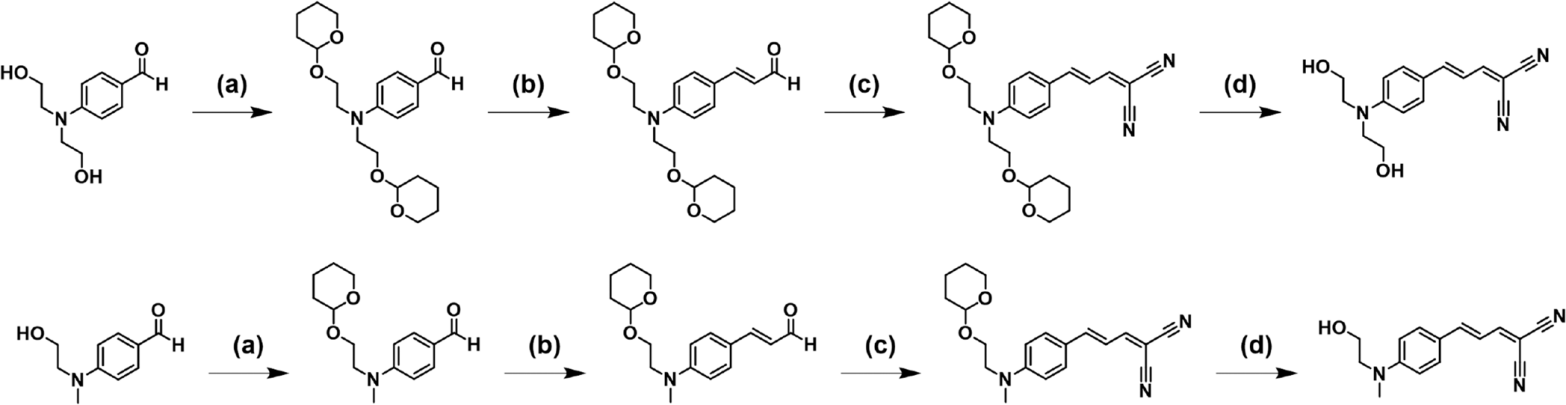
Synthetic route for DANIR-2b­(2OH) (top) and DANIR-2b­(1OH) (bottom). (a) 3,4-Dihydro-2H-pyran, pyridinium toluene sulfonate, DCM, at 40 °C, (b) (1,3-dioxan-2-yl-methyl)­triphenylphosphonium bromide, 18-crown-6, NaH, THF at 20 °C, (c) malononitrile, EtOH at 20 °C, and (d) 1 M HCl, EtOH at 20 °C.

The additional hydroxyl group proved to increase the solubility of the dye in water as DANIR-2b­(2OH) was determined to be soluble up to 867 ± 3 μM, compared to the lower solubility value of 451 ± 6 μM of DANIR-2b­(1OH) (Figure S15).

### Photophysical Characterization

We first characterized the photophysical properties of both DANIR-2b­(1OH) and DANIR-2b­(2OH) in water, as full characterization in aqueous solution has been lacking. The extinction coefficients are comparable: (2.6 ± 0.1) × 10^4^ cm^–1^ M^–1^ at 491 nm for DANIR-2b­(1OH) and (2.8 ± 0.2) × 10^4^ cm^–1^ M^–1^ at 483 nm for DANIR-2b­(2OH) (Figure S16). The value of the extinction coefficient of DANIR-2b­(1OH) in water is within a factor of 2 of the value reported by Watanabe et al. in chloroform.^38^ These values are slightly lower but in the same order of magnitude as ThT (3.6 × 10^4^ cm^–1^ M^–1^ at 412 nm).^18^ However, DANIR-2b­(2OH) exhibits a higher Φ_f_ in water (0.65%) compared to that of DANIR-2b­(1OH) (0.42%) (Figure S17).

To probe their environment sensitivity, we measured absorption and emission spectra in a series of solvents of varying polarity (Figure 3a,b; full series in Figure S18). Both dyes show a broad absorption peak with the maximum in the 470–500 nm region, with a small shoulder in the blue, that shifts with a change in polarity. The emission spectra are featureless (meaning that they do not present multiple peaks due to, for example, a vibronic progression) and vary significantly in the wavelength of emission maxima, depending on the polarity of the solvent. This indicates that emission originates from a charge transfer state, which is consistent with what has been predicted by our ab initio calculations and simulations on similar compounds.^49^

**Figure 3:**
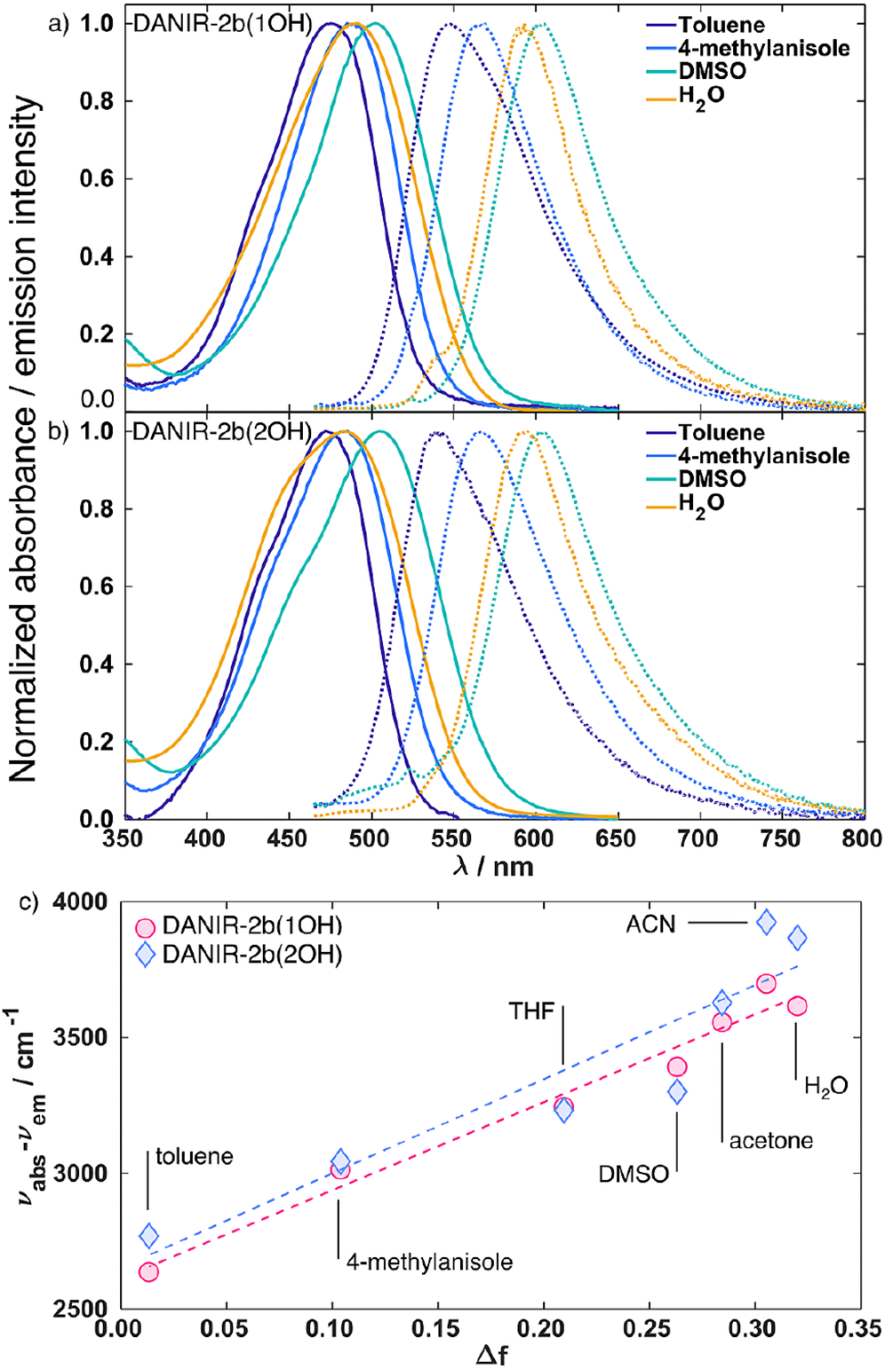
Normalized absorption (solid) and emission (dashed) spectra of (a) DANIR-2b­(1OH) and (b) DANIR-2b­(2OH) in representative solvents: toluene, 4-methylanisole, DMSO, and water. All of the samples were excited at 450 nm. (c) Lippert-Mataga plot of DANIR-2b­(1OH) and DANIR-2b­(2OH). THF: tetrahydrofuran, DMSO: dimethyl sulfoxide, ACN: acetonitrile.

In order to understand the dependence of the emission spectrum on the solvent polarity we analyzed the UV–vis absorption and emission data using the Lippert-Mataga method.^50,51^ The analysis (Figure 3c) reveals a linear dependence of the Stokes shift on the orientation polarizability (Δ*f*) for both dyes. This trend indicates that solute–solvent interactions are primarily determined by the bulk dielectric properties of the solvent rather than specific interactions.

We also attempted to characterize the lifetimes of DANIR-2b­(1OH) and DANIR-2b­(2OH) in water via time-correlated single photon counting (TCSPC). The measurements in water showed that the fluorescence lifetimes for both free dyes are below our instrument response function (0.9 ns). This is consistent with the low Φ_f_ that, based on the Strickler–Berg equation,^52^ corresponds to an estimated lifetime of 20 ps.

### Monitoring Amyloid Aggregation

#### Comparison of Mono- and Bis­(2-hydroxyethyl) DANIR Derivatives

We first evaluated the applicability of the newly synthesized dyes for tracking the aggregation kinetics of amyloid formation. We selected the human Islet Amyloid Polypeptide (hIAPP) as our test case (sequence in Figure 4a). A critical first step in this evaluation is to determine whether the dyes themselves influence the aggregation outcome. Specifically, we needed to check whether they inhibit or accelerate the aggregation process in a concentration-dependent manner and whether they are capable of reproducing the sigmoidal kinetic curve typical of nucleation-based aggregation.

To test this, the kinetics were measured at three different dye concentrations: 2.5 μM, 5.0 μM, and 8.3 μM. The excitation wavelength was set to 490 nm, and the emission was measured at 590 nm. All kinetic assays were run in triplicate, and the results are presented in Figure 4.

**Figure 4:**
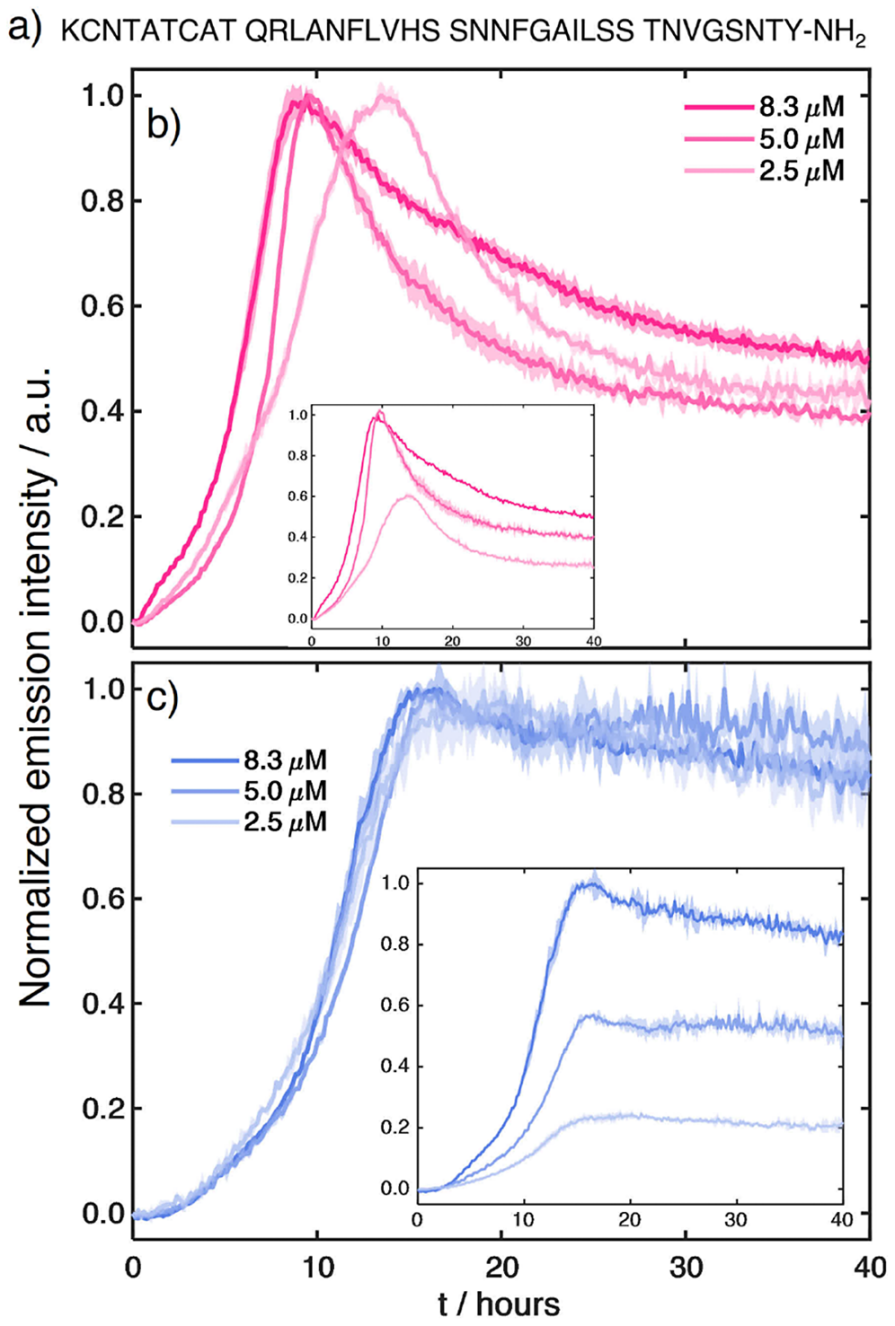
(a) Amino acid sequence of human IAPP. (b) Aggregation kinetics of 100 μM hIAPP in 20 mM Tris (pH 7.4) measured using DANIR-2b­(1OH) at 2.5, 5.0, and 8.3 μM. Excitation: 490 nm; emission: 590 nm. (c) Corresponding aggregation kinetics recorded with DANIR-2b­(2OH) at the same concentrations. Data represent the mean of triplicate wells; shaded regions indicate the standard deviation. The insets in parts (b) and (c) show the kinetic traces normalized with respect to the sample containing the highest concentration of dye.

We observed a drastic difference between the aggregation profiles tracked with DANIR-2b­(1OH) and DANIR-2b­(2OH). In the former case, the aggregation shows significant variations at different concentrations, with a reduced lag phase as the dye concentration increases (Figure 4b). A dramatic change in the intensity profile is also observed: the fluorescence intensity appears to reach its maximum around the 10 h mark, after which it decays nonlinearly to a fraction of its peak intensity. Although some intensity drops are often seen in ThT assays, a reduction of 50% of the intensity of the signal is rare. This likely indicates that the dye undergoes reorientation or dissociates from the fibril surface as aggregation progresses and more fibrils form.

On the other hand, DANIR-2b­(2OH) exhibits fully reproducible kinetic traces at all concentrations tested, with no variations in the length of the lag or elongation phases (Figure 4c). Although a small drop in intensity is observed over the extended run, it amounts to at most 10% of the maximum fluorescence, and it is comparable to what has been observed in many ThT assays.^53–58^ These results demonstrate that DANIR-2b­(2OH) does not interfere with the aggregation process and is a more reliable compound for these assays. The most likely explanation for the different behavior is the 2-fold higher water solubility of DANIR-2b­(2OH) that prevents it from excessively interacting with the protein aggregates or self-association in an aqueous environment. The different effects of the two dyes on the aggregation of hIAPP were also observed by atomic force microscopy (Figure S19): the samples aggregated in the presence of ThT and DANIR-2b­(2OH) are qualitatively similar. Meanwhile, the sample aggregated in the presence of DANIR-2b­(1OH) appears different in terms of the distribution and length of the fibrils. Based on this superior and nonperturbative performance, all subsequent investigations and discussion will focus exclusively on DANIR-2b­(2OH).

To further verify the applicability of DANIR-2b­(2OH) as an aggregation probe, we measured the aggregation kinetics of Aβ_42_ and insulin. The kinetic traces (Figure 5) clearly show the typical sigmoidal profile, indicating that this dye is able to follow the formation of amyloid fibrils of these proteins, as well. This demonstrates a broader applicability of DANIR-2b­(2OH), not just limited to the case of hIAPP.

**Figure 5:**
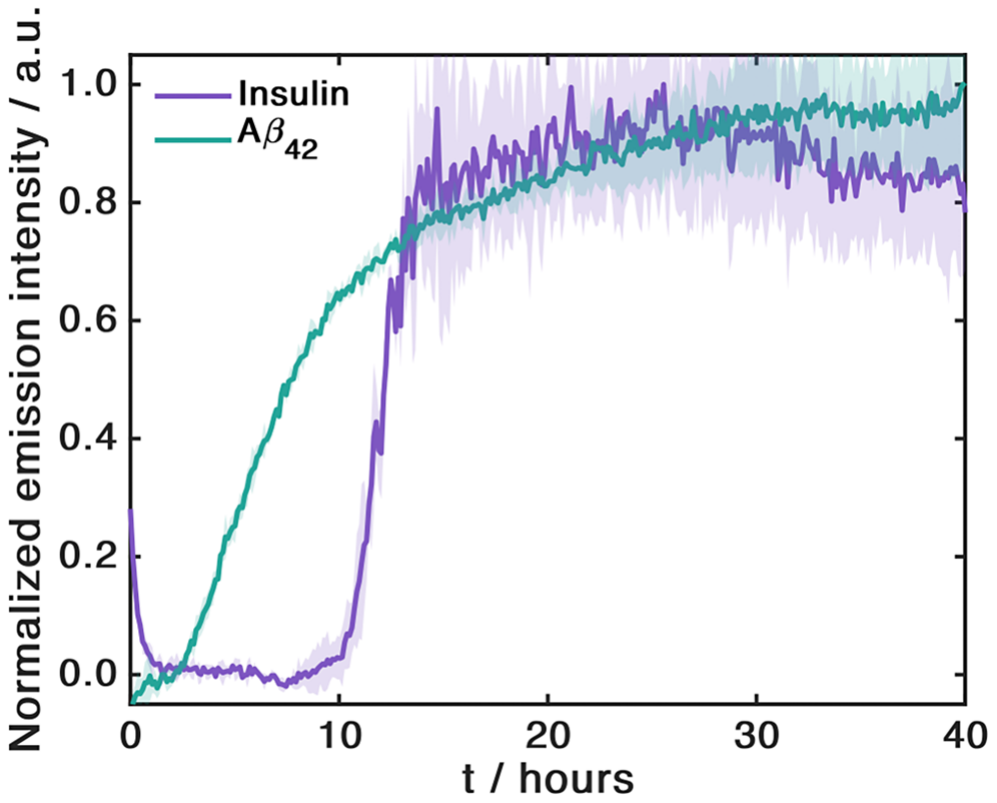
Aggregation kinetics of 50 μM insulin and 50 μM Aβ_42_ in 20 mM Tris (pH 7.4) monitored by 4 μM DANIR-2b­(2OH). Data represent the mean of duplicate wells; shaded regions indicate the standard deviation.

Given that aggregation assays can run for hours or days, we conducted photostability tests to verify whether this dye can withstand prolonged exposure to the lamp of the plate reader, as required for these experiments. The samples do not show a significant sign of degradation over 150 h, indicating that it presents an adequate level of photostability to be used to track amyloid fibril aggregation kinetics (Figure S20). To further characterize the interaction between DANIR-2b­(2OH) and hIAPP fibrils, we measured the aggregation kinetics of 100, 50, and 20 μM hIAPP in the presence of 4 μM DANIR-2b­(2OH) (Figure S21). The results show the fluorescence signal increases proportionally in intensity when comparing the 50 μM to the 20 μM one. However, the signal intensity of the 100 μM sample is comparable to the 50 μM one, indicating that the saturation point has been reached. This was confirmed by the signal increasing when repeating the aggregation kinetics experiment of 100 μM of hIAPP in the presence of 8 μM of DANIR-2b­(2OH). Based on these results, it is possible to conclude that DANIR-2b­(2OH) can be used for the quantification of fibrils, given that the protein:dye concentration ratio is lower than 12.5. Previous results of ThT-based assays for the quantification of hIAPP fibrils were performed with an excess concentration of dye compared to the monomeric protein, indicating that DANIR-2b­(2OH) represents a valid alternative to ThT as it yields comparable results at lower working concentrations.^59^

#### Full Spectral Kinetics and Fluorescence Lifetimes of hIAPP Aggregation

To understand the behavior of the dye during aggregation, we moved beyond single-wavelength readings and measured the full time-dependent fluorescence spectra of DANIR-2b­(2OH) in the presence of 100 μM hIAPP. This approach allows us to identify not only intensity changes but also any shifts in peak position or spectral shape. The resulting spectra are presented in Figure 6. As shown in Figure 6a, we observed a significant enhancement of the DANIR-2b­(2OH) emission over the 35-h measurement. Normalizing the spectra (Figure 6b) clearly reveals a blue-shift of the emission peak of approximately 15 nm. Overall, the behavior resembles that of a typical aggregation probe, with weak emission in the presence of a monomeric peptide and strong emission as aggregation proceeds.

**Figure 6:**
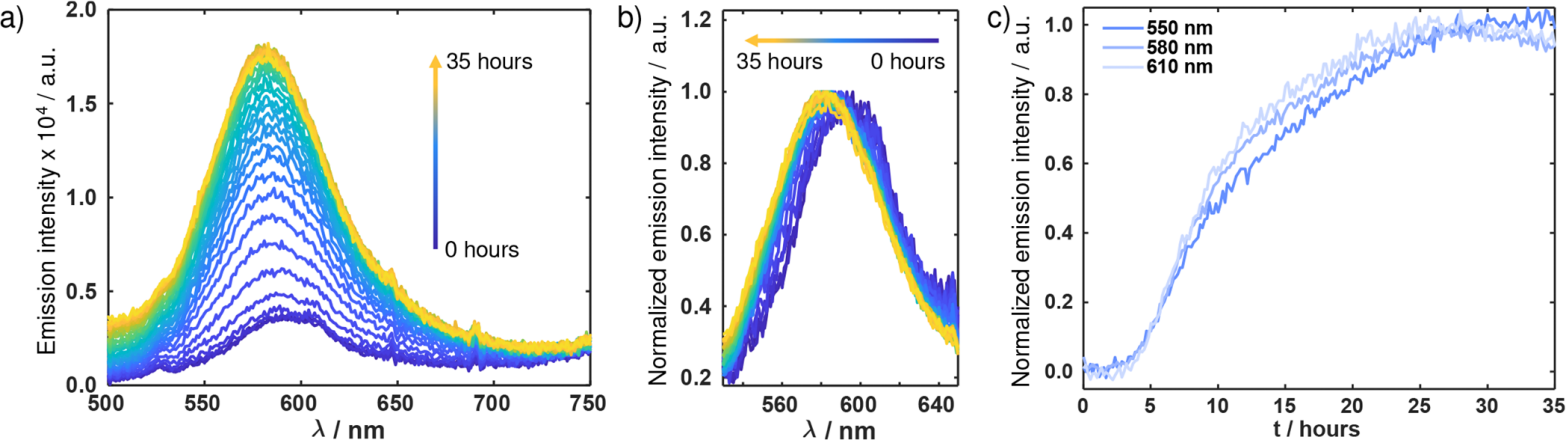
(a) Emission spectra of 8 μM DANIR-2b­(2OH) in the presence of 100 μM hIAPP in 10 mM HEPES (pH 7.4) recorded hourly. (b) Normalized emission spectra showing a progressive blue shift during aggregation. (c) Kinetic traces extracted from panel (a) at 550, 580, and 610 nm.

The kinetic traces extracted from the full spectral data (Figure 6a) are shown in Figure 6c for three representative wavelengths: 550, 580 (the peak maximum), and 610 nm. All three traces reproduce the expected sigmoidal curve and, when normalized, are nearly superimposable. This confirms that probing at different wavelengths across the emission band yields identical kinetic profiles with no variations in the lag phase. This offers practical flexibility as monitoring at the 580 nm peak is not strictly required.

Interestingly, the binding to amyloid fibrils also has a dramatic effect on the fluorescence lifetime. Our TCSPC results (Figure S22) show that when bound to fibrils, the lifetime of DANIR-2b­(2OH) increases to 1.5 ns. This is a significant change from the free dye in water, which has an estimated lifetime in the tens of picoseconds, and is long enough to be easily quantified on standard TCSPC instruments. This suggests that the fluorescence lifetime could be used as an alternative reporter for amyloid binding, although further work is needed to verify if structurally sensitive information can be extracted from such measurements.

Additionally, having characterized the interaction between hIAPP fibrils and DANIR-2b­(2OH), we tested whether this dye retained the two-photon absorption properties of the parent compound. INS1-E cells stained with DANIR-2b­(2OH), excited at 980 nm, clearly showed fluorescence from the dye, confirming that it is suitable for both one- and two-photon fluorescence imaging experiments (Figure S23). This cell line was chosen as it is commonly used for mechanistic studies on the effects of hIAPP on pancreatic cells.^60–62^

#### Benchmarking against ThT and Cryo-EM Analysis

We proceeded to benchmark DANIR-2b­(2OH) against ThT to evaluate whether both dyes report on the same aggregation events and, more importantly, whether DANIR-2b­(2OH) can detect species invisible to standard ThT assays. To minimize the risk of well-to-well variability, we measured the aggregation with both ThT and DANIR-2b­(2OH) present in the same sample well. While some competitive binding might be expected, we kept the dyes at low enough concentrations (8.3 μM) relative to the peptide (16 μM) to ensure a large excess of available binding sites for both probes. The results of this assay, shown in Figure 7a, reveal significant differences between the two dye signals. The most immediate difference is the lag phase duration. The kinetic trace for DANIR-2b­(2OH) shows a lag phase of only 1.8 h, whereas the ThT signal has a lag phase of 4.5 h. Furthermore, the DANIR-2b­(2OH) signal displays a rapid elongation phase and essentially plateaus by 15 h. In contrast, the ThT signal rises much more slowly and continues to increase over the entire 40-h experiment.

**Figure 7:**
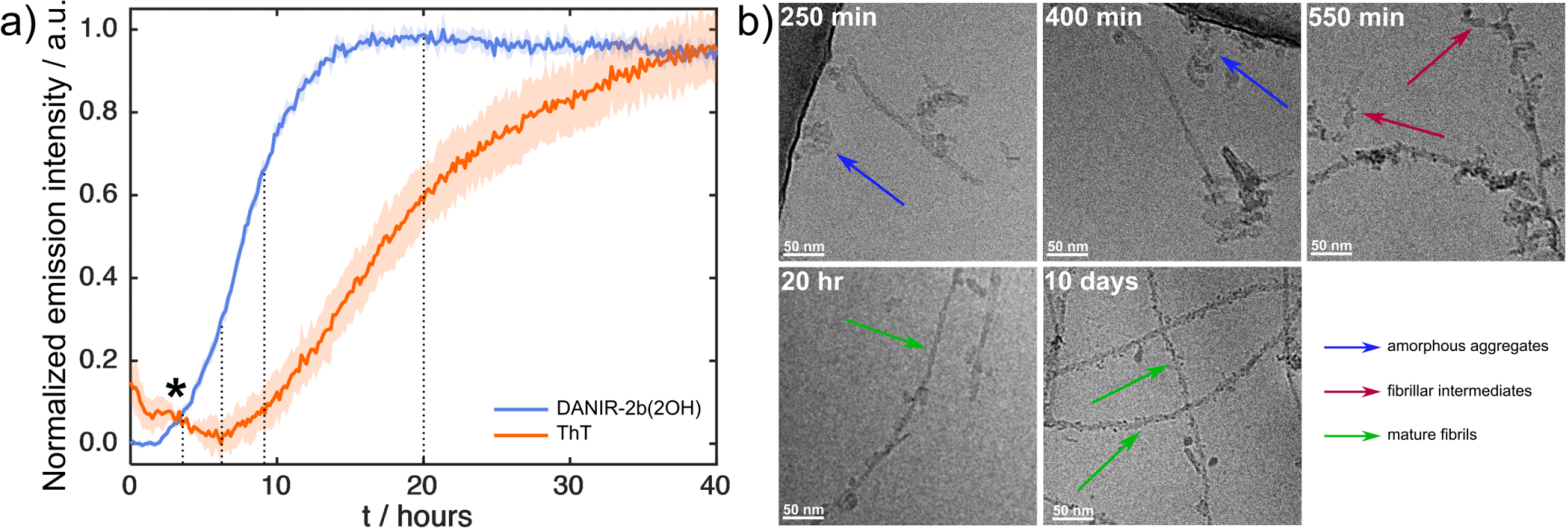
(a) Aggregation kinetics of 16 μM hIAPP in 20 mM Tris (pH 7.4) monitored by 12 μM ThT and 4 μM DANIR-2b­(2OH) fluorescence at 490 and 590 nm, respectively. Data represent the mean of triplicate wells; shaded regions indicate standard deviation. The initial decay in the ThT traces marked with an asterisk is caused by an instrument artifact. Vertical dashed lines indicate the time points at which samples were aliquoted for Cryo-EM analysis. (b) Cryo-EM micrographs of hIAPP fibrils taken at the time points indicated by the dashed lines in panel (a).

This discrepancy suggests that DANIR-2b­(2OH) is sensitive to prefibrillar species that ThT does not detect. This behavior is similar to that reported for certain olygothiophenes, which can bind early aggregates of Aβ_1–40_, or the one of Thioflavin X, which can bind early aggregates of α-synuclein.^28,63^ To verify what structures DANIR-2b­(2OH) is binding to, we performed time-resolved cryo-electron microscopy (cryo-EM) by aliquoting samples at key time points from the kinetic assay (Figure 7b). These time points were chosen based on the kinetic traces. The first time point was at 250 min (4.2 h), where the DANIR-2b­(2OH) signal is rising, but ThT is still in its lag phase. The second was at 400 min (6.7 h), with DANIR-2b­(2OH) in its fast elongation phase, while ThT was just beginning to show a signal. The third point was at 550 min (9.2 h), where the DANIR-2b­(2OH) signal was at >60% of its maximum, while the ThT signal was still very low. The final time point was at 20 h, when the DANIR-2b­(2OH) signal had plateaued, while ThT was still rising.

The cryo-EM micrographs (Figure 7b) confirm the hypothesis that DANIR-2b­(2OH) can bind to prefibrillar structures. At 250 min, the grids already show small fibrillar structures, some amorphous aggregates, and small oligomeric species. These are the structures that DANIR-2b­(2OH) is clearly detecting, while ThT is not. At 400 and 550 min, these fibrils become longer and more abundant. By the 20 h mark, the grids show a significant population of mature amyloid fibrils, with reduced amorphous material. These micrographs are very similar to samples left to aggregate for 10 days, which aligns with the DANIR-2b­(2OH) kinetic trace reaching its plateau. These results demonstrate that DANIR-2b­(2OH) is capable of binding to early-stage fibrillar species that are effectively undetectable by a conventional ThT assay.

#### Performance with ThT-Negative Amyloids

Another important practical observation from Figure 7a is the drastically better signal stability for DANIR-2b­(2OH) compared to ThT. ThT assays are often noisy, with significant deviations, as seen in the large error bars for the ThT trace.^34,64–66^ Additionally, from Figure S24, it can be seen that DANIR-2b­(2OH) presents a 6-fold higher signal-to-noise ratio (S/N) compared to ThT (6.8 and 1.2, respectively). This encouraged us to test DANIR-2b­(2OH) on an amyloid system where ThT is known to fail. We chose the pufferfish (*Takifugu rubripes*) IAPP (pfIAPP), which has been shown to form fibrils visible by EM but produces negligible ThT fluorescence.^23^

We synthesized pfIAPP (sequence in Figure 8a) and carried out the aggregation assays. In preliminary tests, we found that at high concentrations (>50 μM), pfIAPP aggregation could be detected by ThT (data not shown). However, this signal was lost at lower concentrations, suggesting that the ThT-resistant polymorphs form under these conditions. We therefore set our final pfIAPP concentration to 16 μM to test the dyes under conditions invisible to standard ThT assays. The results are presented in Figure 8b. The kinetic trace measured with ThT is barely distinguishable from the baseline noise, while the trace measured with DANIR-2b­(2OH) is clearly visible and shows a reproducible sigmoidal curve. The improved signal quality is quantified in Figure 8c, which shows that the standard deviation for the DANIR-2b­(2OH) signal is nearly 2 orders of magnitude lower than that of ThT. Furthermore, the lag phase for pfIAPP aggregation measured with DANIR-2b­(2OH) is again shorter than that measured with ThT, demonstrating that the sensitivity of DANIR-2b­(2OH) to early species is not limited to just hIAPP. Moreover, the results demonstrate the ability of DANIR-2b­(2OH) to detect fibrils even when the fluorescent signals are extremely weak. This creates new opportunities for measuring peptide aggregation at concentrations significantly lower than those studied to date.

**Figure 8:**
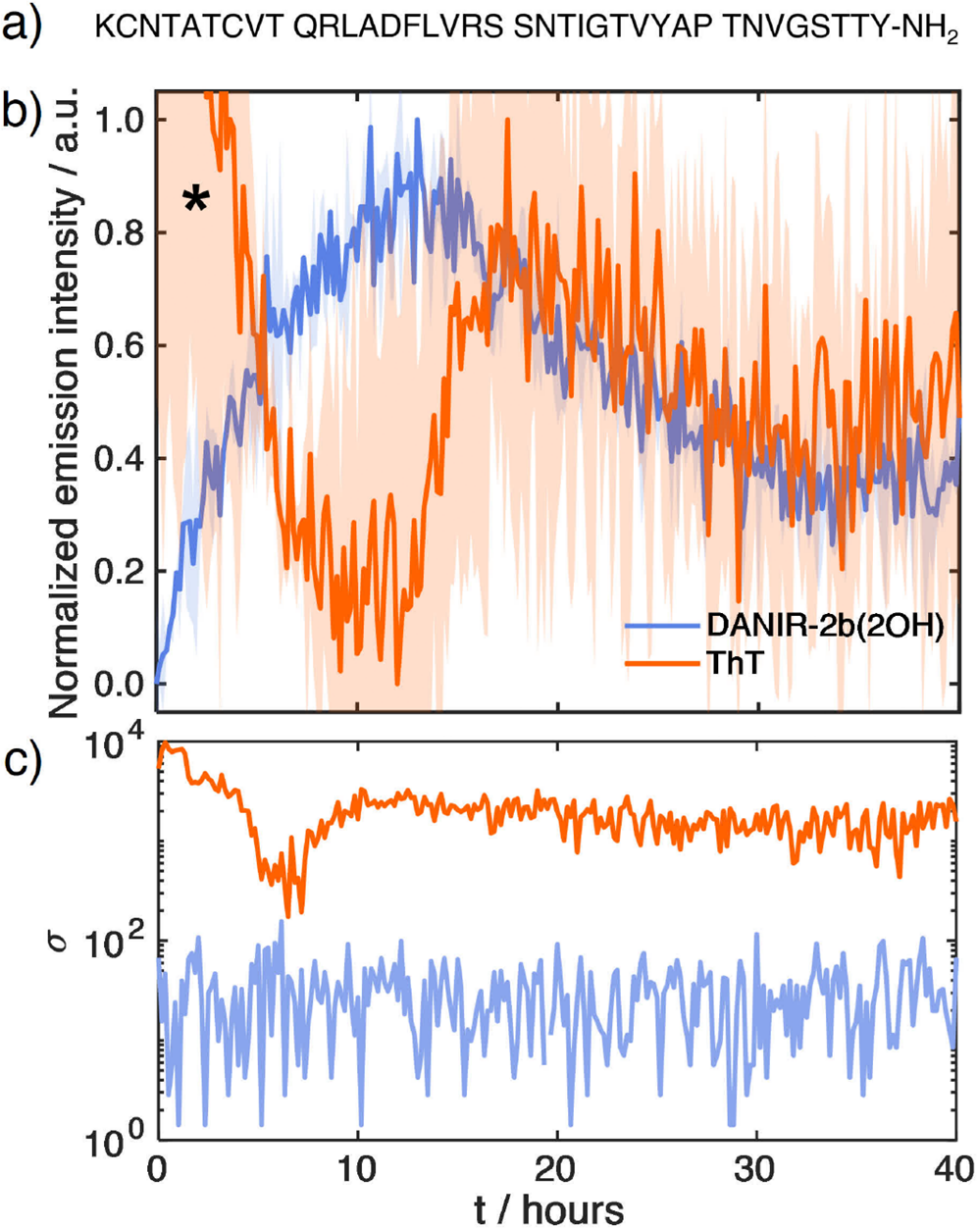
(a) Amino acid sequence of 16 μM pfIAPP. (b) Aggregation kinetics of 16 μM pfIAPP in 20 mM Tris (pH 7.4) monitored by 12 μM ThT and 4 μM DANIR-2b­(2OH) fluorescence and (c) corresponding standard deviations. Data represent the mean of duplicate wells; shaded regions indicate standard deviation. The initial decay in the ThT traces marked with an asterisks is caused by an instrument artifact.

## Discussion

Based on our results, the photophysical mechanism of fluorescence enhancement for DANIR-2b­(2OH) appears to be fundamentally different from that of ThT, which explains their different performances in kinetic assays. The activation of ThT fluorescence is well-understood to result from the restriction of intramolecular rotation around its central C–C bond upon binding.^21^ This mechanism requires a binding site that is sufficiently rigid and is sterically confined. DANIR-2b­(2OH), as a push–pull dye, does not appear to rely on this specific pathway. Instead, it has been suggested that deactivation in DANIR dyes can occur via a conical intersection involving a trans–cis isomerization of the double bond chain, with the activation energy for such a process scaling with the number of double bonds in the system.^49^

Its enhanced emission is consistent with a response to a change in the local environment, specifically increased rigidity. When free in aqueous solution, the dye has a low Φ_f_ (0.65%) and an extremely short fluorescence lifetime. Upon binding to hIAPP fibrils, we observed a significant increase in the fluorescence lifetime to 1.5 ns, which is an indication of immobilization within a binding pocket. Based on the Strickler–Berg equation, it is possible to estimate the value of Φ_f_ as 39% for the bound dye, meaning that binding to fibrils enhances Φ_f_ by 2 orders of magnitude.^52^ The change of environment is further supported by the 15 nm blue-shift in its emission spectrum during aggregation, which is typical for push–pull dyes moving from a polar solvent to a more nonpolar, rigid binding site.

The mechanistic differences in the deactivation pathways for the excited states of ThT and DANIR-2b­(2OH) can also provide a clear explanation for the kinetic data in Figure 7. ThT likely requires the formation of mature, well-ordered β-sheets to become sufficiently constrained for fluorescence enhancement, as deactivation involves rotation around the single bond between the two rings. Meaning that only a tight binding pocket can prevent it. These sites are apparently absent in the early stages, which makes ThT give a zero signal essentially. Meanwhile, as the deactivation of DANIR-2b­(2OH) involves a trans–cis isomerization, with a much larger structural change than for ThT, it can be prevented by larger binding sites that are present in more flexible or less-ordered structures, such as small oligomers or protofibrils. Our time-resolved cryo-EM data strongly support this. At 250 min, when the DANIR-2b­(2OH) signal is already rising, but the ThT signal is flat, we clearly observe the presence of small fibrillar structures and oligomeric species. ThT begins to report a signal only once these fibrils become longer and more abundant. This confirms that the shorter lag phase of DANIR-2b­(2OH) is due to its ability to detect early-stage aggregates that are invisible to ThT.

When it comes to the comparison of the signal quality of ThT and DANIR-2b­(2OH), the pfIAPP aggregation experiment constitutes a key result. In this case, both dyes show a drastically reduced signal, though the superior signal quality of DANIR-2b­(2OH) makes the kinetic trace discernible (Figure S24). This suggests that the specific fibril polymorph formed by pfIAPP lacks efficient binding sites, whether these are rigid grooves for ThT or appropriate hydrophobic/immobilizing sites for DANIR-2b­(2OH). The problem in this case is not the fluorescence mechanisms but rather an inefficient binding affinity for this particular fibril morphology. This indicates that although DANIR-2b­(2OH) is more versatile than ThT, its binding is still selective and dependent on fibril polymorphism. This is further corroborated by the observation of the signal drop in Figure 8b, which suggests a change in the fibril structure to one with a lower binding affinity toward DANIR-2b­(2OH).

A significant practical advantage of DANIR-2b­(2OH) over ThT is the dramatic improvement in the signal stability. This is immediately visible in the kinetic traces for both hIAPP (Figure 7a) and pfIAPP (Figure 8b). The shaded error bands, which represent the standard deviation across wells, are consistently and substantially smaller for DANIR-2b­(2OH).

The difference in S/N between kinetic traces depends on the stability of the light source over time, the behavior of the sample, and the responsivity of the detector. It is often noted that photomultiplier tubes, which are common in plate readers, have a higher quantum efficiency in the blue region where ThT emits. If detector sensitivity were the limiting factor, ThT should, in theory, provide a cleaner signal. The fact that we and others consistently observe the opposite, that ThT is significantly noisier, demonstrates that these assays are background-limited, not detector-limited. Additionally, by measuring the signal of ThT and DANIR-2b­(2OH) in parallel from the same wells, it is possible to exclude variability due to sample behavior and fluctuations in lamp power. Thus, the high background from spectral bleed-through and scattering is likely the dominant source of the noise.

ThT is characterized by a relatively smaller Stokes shift when bound to amyloid fibrils.^67^ A study on ThT binding to insulin fibrils indicated that the emission band retains its position relative to the free solution; however, the absorption band shifts to the red, effectively reducing the Stokes shift and increasing the overlap.^68^ This drastically increases the chances of cross-talk due to overlapping spectral tails. The analysis of numerous aggregation kinetics shows that the signal fluctuation magnitude increases for later stages of aggregation when a significant population of amyloids forms.^53–58^ This observation is consistent with the increasing extent of spectral overlap for bound ThT versus the free dye in solution. The effect becomes negligible for dyes characterized by a large separation of absorption and emission bands, such as the studied DANIR analogs. Although the superior signal stability is evident from our data, it is not possible to compare the S/N performance of DANIR-2b­(2OH) with that of other dyes because error bars and statistical analysis of the kinetics data are generally not provided in the literature.^69–72^ Direct S/N benchmarking across dyes will require standardized, side-by-side measurements that report replicate statistics and full optical bandpass settings, enabling a quantitative placement of DANIR-2b­(2OH) relative to other probes.

## Conclusion

We designed and synthesized DANIR-2b­(2OH), a water-soluble push–pull dye that reports amyloid formation with sensitivity and stability higher than those of ThT in standard plate-reader assays. DANIR-2b­(2OH) detects early aggregate populations that are not captured by ThT and yields reliable signals in a ThT-negative system (pfIAPP). Together with its straightforward synthesis, these properties make DANIR-2b­(2OH) a useful complementary probe for routine aggregation measurements.

In future studies, we aim at expanding benchmarks across other amyloid systems and specific fibril polymorphs. It is also desirable to test the performance in complex matrices, such as phospholipids and crowded solutions. These steps will establish when DANIR dyes can replace and when they should complement ThT in high-throughput kinetic assays and imaging.

## Supplementary Material



## Notes

(1) DFT-derived molecular orbitals of the HOMO and LUMO states; (2) Details on the chemical synthesis of the studied compounds, NMR chemical shifts, and LC/MS characterization; (3) absorption and emission spectra used to determine extinction coefficients, solubility, Lippert-Mataga plot and quantum yields; (4) atomic force microscopy images of mature IAPP fibrils; (5) photostability test; (6) titration experiments; (7) time-correlated single photon counting results; (8) two-photon fluoresce microscopy image of INS1-E cells stained with DANIR-2b­(2OH)­and (9) data for the determination of DANIR-2b­(2OH) and ThT S/N (PDF)
