# Supplementary material for: A Highly Sensitive Water-Soluble Donor–Acceptor Dye for Early-Stage Amyloid Aggregation Kinetics
Source: J Phys Chem B. 2026 Mar 10;130(11):3042–53. doi: 10.1021/acs.jpcb.5c07705 (PMC13006956; doi:10.1021/acs.jpcb.5c07705)
Supplement: Supplementary file 1 [file jp5c07705_si_001.pdf]

Supporting Information: A Highly Sensitive Water-Soluble Donor-Acceptor Dye for Early-Stage Amyloid Aggregation Kinetics

Giorgio Scattolini, Carlos Enrique Torres-Méndez, Dylan Valli, Mikołaj Ignacy Kuska, Nidhi Kaul, Leif Hammarström, Haining Tian, and Michał Maj*

Department of Chemistry – Ångström Laboratory, Uppsala University, Box 523, 751 20 Uppsala, Sweden

E-mail: michal.maj@kemi.uu.se

Contents

1 Molecular orbitals **3**

2 Synthetic procedure **4**

2.1 Chemicals . . . 4

2.2 Synthesis of DANIR-2b(1OH) . . . 4

2.3 Synthesis of DANIR-2b(2OH) . . . 9

3 Solubility measurements **14**

4 Extinction coefficients determination **15**

5 Quantum yields determination **15**

6 Absorption and emission spectra **16**

7 Atomic force microscopy **17**

8 Photostability **17**

9 Titration experiments **18**

10 Time-Correlated Single Photon Counting **19**

11 Two-photon fluorescence microscopy **20**

12 S/N determination **21**

1 Molecular orbitals

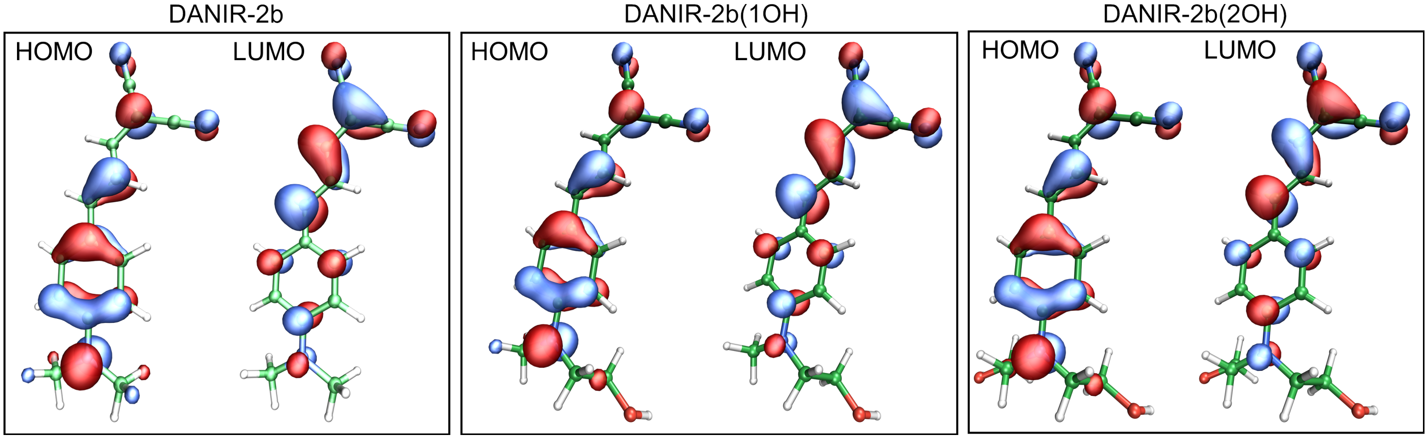

Figure S1: The Highest Occupied Molecular Orbital (HOMO) and Lowest Unoccupied Molecular Orbital (LUMO) of DANIR-2b, DANIR-2b(1OH), and DANIR-2b(2OH). Calculations were performed at the CAM-B3LYP/6-311++G(d,p) level of theory.

2 Synthetic procedure

The two dyes were synthesized by a reported method from the literature with small modifications.[1]

2.1 Chemicals

All the chemicals employed were obtained from commercial suppliers and were used as received without further purification. ^{1}H spectra were collected with a Jeol Resonance 400 MHz spectrometer at 293 K. The following abbreviations were used to indicate multiplicity: s (singlet), d (doublet), t (triplet), dd (doublet of doublets) and ddd (doublet of doublets of doublets). Chemical shifts (δ) are expressed in ppm using the residual signal of the acetonitrile solvent as internal standard (δ = 1.94 ppm for ^{1}H spectra). Coupling constants (J) are expressed in Hertz (Hz).

2.2 Synthesis of DANIR-2b(1OH)

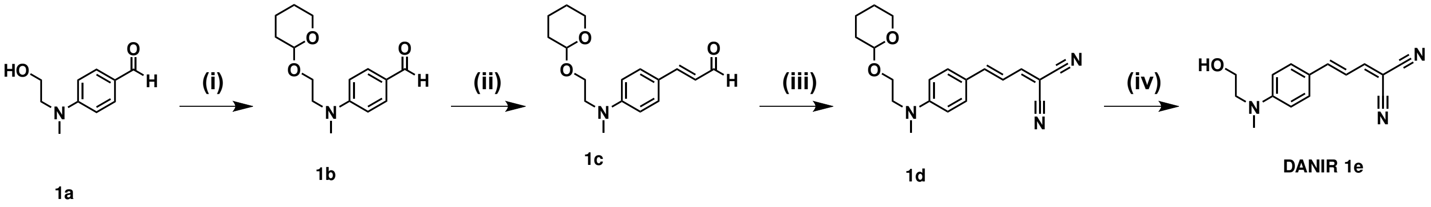

Figure S2: (i) 3,4-dihydro-2H-pyran, pyridinium toluene sulfonate, DCM at 40 °C (ii) (1,3-dioxan-2-yl-methyl)triphenylphosphoniumbromide,18-crown-6, NaH, THF at 20 °C (iii) Malononitrile, EtOH at 20 °C (iv) 1 M HCl, EtOH at 20 °C

In a 40 mL crim vial loaded with a stir bar, 1.14 g (6.4 mmol) of 4-((2-hydroxyethyl)(methyl)amino)benzaldehyde (**1a**) are dissolved in 20 mL of anhydrous dichloromethane (DCM). To this mixture, 2.0 g (24 mmol) of 3,4-dihydro-2H-pyran and 400 mg (1.6 mmol) of pyridinium toluene-4-sulphonate are added. The reaction is stirred at 40° C for 3 hours and then cooled to room temperature. The mixture is filtered over silica and excess solvent is removed under vacuum. A yellow oil consisting of 4-(methyl(2-((tetrahydro-2H-pyran-2-yl)oxy)ethyl)amino)benzaldehyde (**1b**) is obtained (1.7 g). LC-MS (ESI) m/z: Found 264.10; calculated for $[M+H]^{+}$: 264.16. Rt= 4.84 minutes.

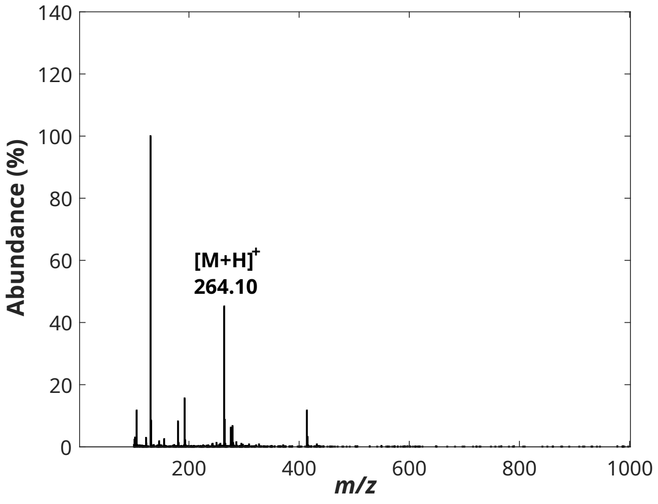

Figure S3: Mass spectrum of **1b**.

A 3-neck round bottom flask was attached to a condenser under a positive flow of argon. A stirred suspension of 1.7 g of **1b** (6.5 mmol) is prepared with 75 mL of anhydrous THF. Following this, (1,3-dioxolan-2-ylmethyl)triphenylphosphonium bromide (6.05 g, 14 mmol) and 18-crown-6 (26 mg, 0.1 mmol) are added, the system is closed and the mixture is stirred for another 30 minutes. The third neck of the flask was opened under a positive flow of argon and NaH (60% dispersion in mineral oil, 2.5 g) was added in small portions over the course of 20 minutes. The flask was closed again and the mixture was stirred for 3 hours at room temperature. The reaction was quenched with 4% water in THF until no more gas evolution was observed. The mixture was extracted with AcOEt (3×100 mL). The combined organic phase was dried over anhydrous sodium sulfate and the solvent was removed under vacuum, the residue was purified by silica gel chromatography (Toluene:EtOAc = 3:1) to give a yellow oily liquid consisting of (E)-3-(4-(methyl(2-((tetrahydro-2H-pyran-2-yl)oxy)ethyl)amino)phenyl)acrylaldehyde (**1c**) (1.8g). LC-MS (ESI) m/z: Found 290.20; calculated for $[M+H]^+$: 290.17. Rt= 5.50 minutes.

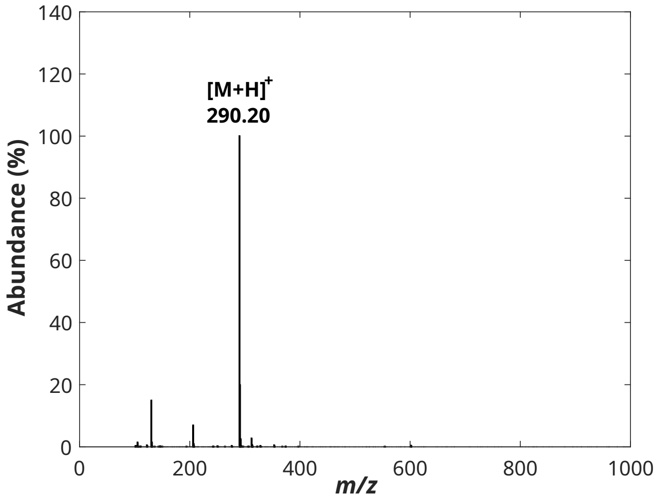

Figure S4: Mass spectrum of **1c**.

A round bottom flask is loaded with 3.6 g of malononitrile (55 mmol) are added to a solution of 1.16g of **1c** (4 mmol) in EtOH (100 mL), no catalyst was needed for this Knoevenagel condensation. The mixture was stirred overnight at room temperature. The solvent was removed in a vacuum. The residue was purified by silica gel chromatography (Heptane:THF = 1:1) to give a red viscous oil consisting of **1d**. The oil is dissolved in 2% HCl in EtOH and stirred at room temperature for 3 hours. The mixture is neutralized with K_2CO_3, filtered and concentrated under vacuum. The residue was purified by silica gel chromatography (diethyl ether:iPrOH = 9:1) to give a red viscous oil consisting of (E)-2-(3-(4-((2-hydroxyethyl)(methyl)amino)phenyl)allylidene)malononitrile (1e). LC-MS (ESI) m/z: Found 254.10; calculated for $[M+H]^+$: 254.12. Rt= 4.76 minutes. 1H NMR (400 MHz, CD_3CN) δ: 7.71 (d, J = 11.8 Hz, 1H), 7.53 (d, J = 8.9 Hz, 2H), 7.32 (d, J = 14.8 Hz, 1H), 6.99 (dd, J = 14.8, 11.8 Hz, 1H), 6.77 (d, J = 9.1 Hz, 2H), 3.68 (dd, J = 10.6, 5.4 Hz, 2H), 3.54 (t, J = 5.8 Hz, 2H), 3.07 (s, 3H).

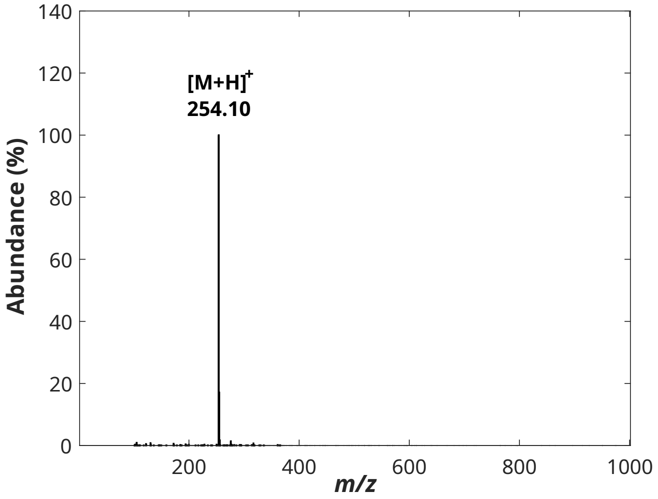

Figure S5: Mass spectrum of **1e**.

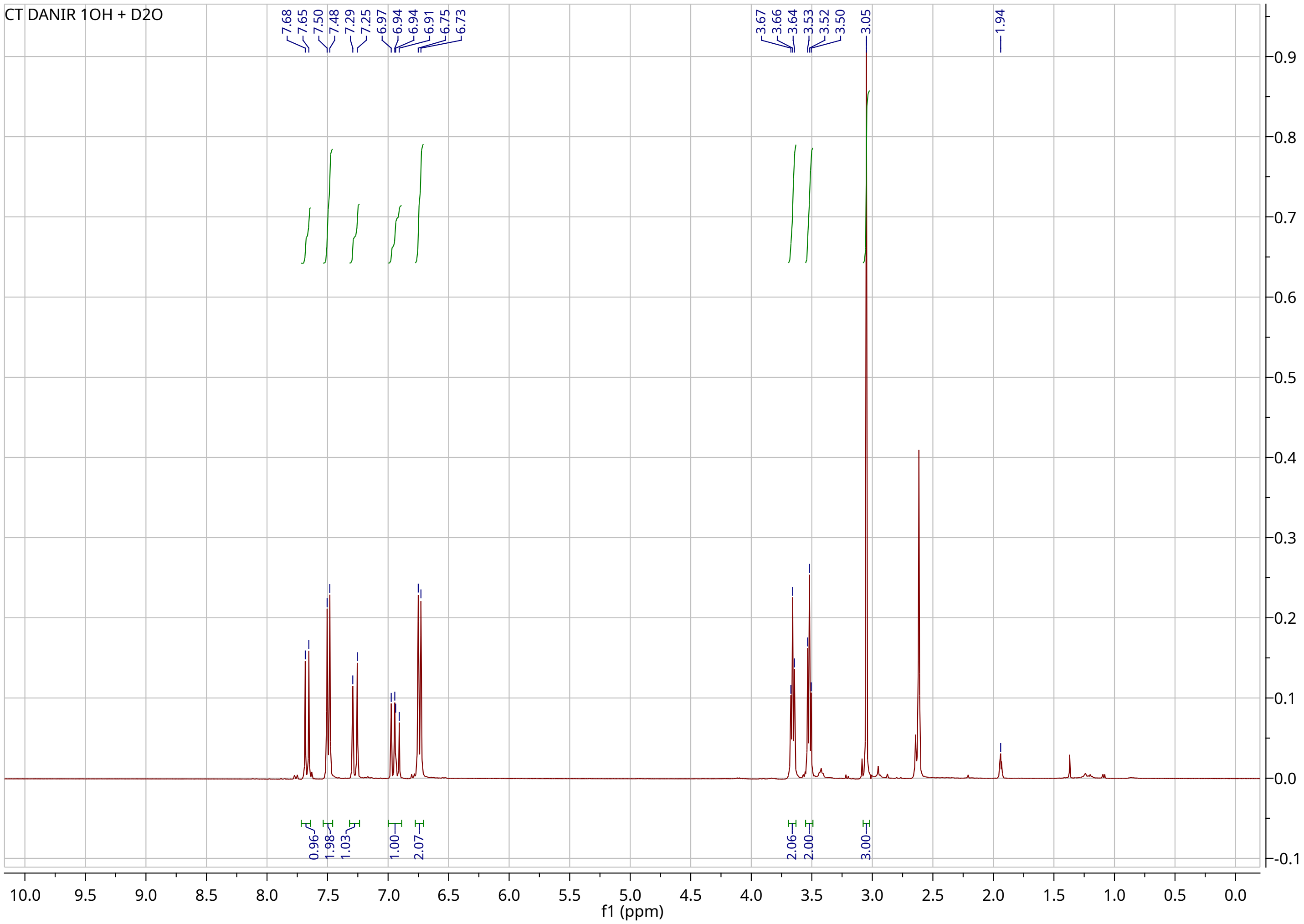

Figure S6: ^{1}H NMR spectra of DANIR-2b(1OH) in CD_3CN/D_2O 99:1.

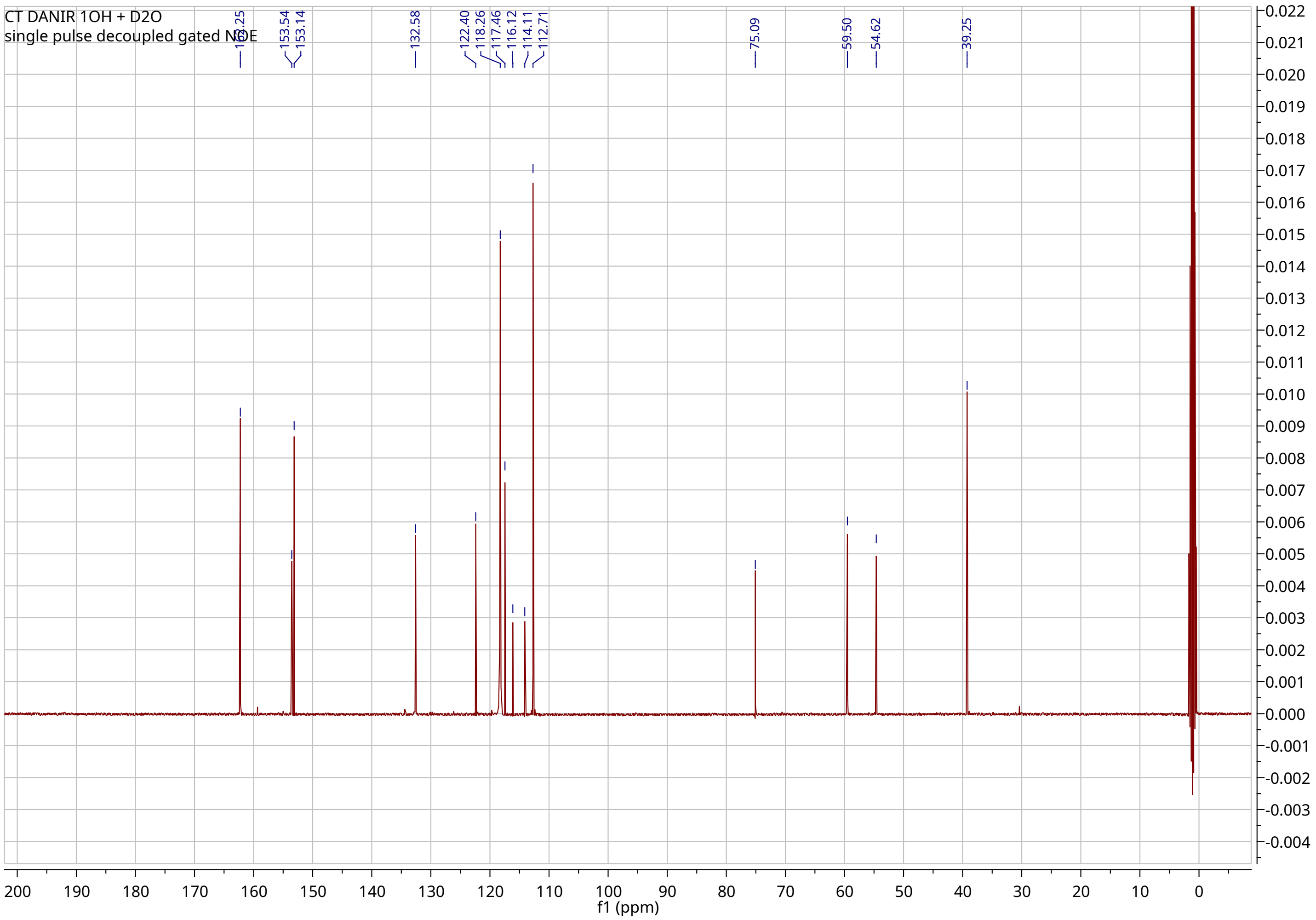

Figure S7: ^{13}C NMR spectra of DANIR-2b(1OH) in CD_3CN/D_2O 99:1.

2.3 Synthesis of DANIR-2b(2OH)

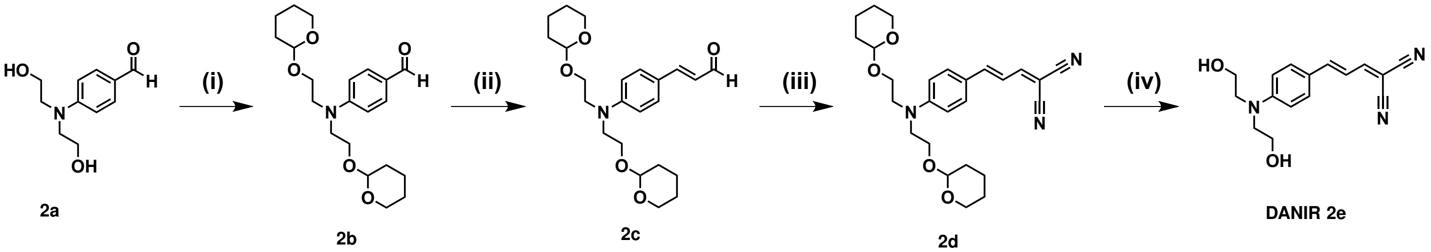

Figure S8: (i) 3,4-dihydro-2H-pyran, pyridinium toluene sulfonate, DCM at 40 °C (ii) (1,3-dioxan-2-yl-methyl)triphenylphosphoniumbromide,18-crown-6, NaH, THF at 20 °C (iii) Malononitrile, EtOH at 20 °C (iv) 1 M HCl, EtOH at 20 °C

In a 40 mL crim vial loaded with a stir bar, 1.14 g (6.4 mmol) of 4-(bis(2-hydroxyethyl)amino)benzaldehyde (2a) are dissolved in 20 mL of anhydrous dichloromethane (DCM). To this mixture, 2.0 g (24 mmol) of 3,4-dihydro-2H-pyran and 400 mg (1.6 mmol) of pyridinium toluene-4-sulphonate are added. The reaction is stirred at 40° C for 3 hours and then cooled to room temperature. The mixture is filtered over silica and excess solvent is removed under vacuum. A yellow oil consisting of 4-(bis(2-((tetrahydro-2H-pyran-2-yl)oxy)ethyl)amino)benzaldehyde (2b) is obtained (1.7 g). LC-MS (ESI) m/z: Found 378.20; calculated for $[M+H]^+$: 378.23. Rt= 5.96 minutes.

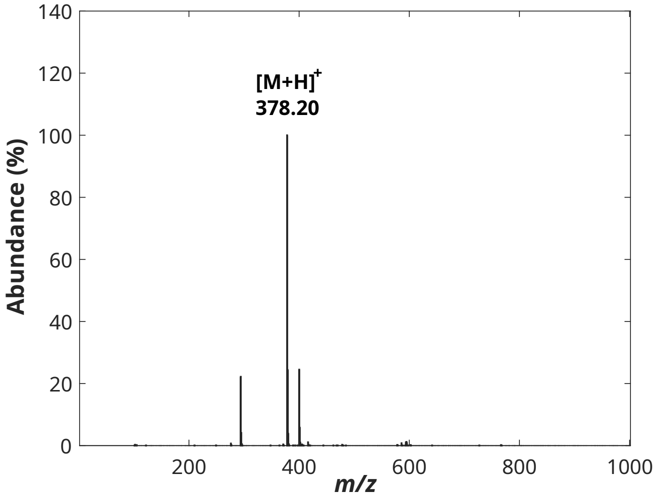

Figure S9: Mass spectrum of **2b**.

A 3-neck round bottom flask was attached to a condenser under a positive flow of argon. A stirred suspension of 1.7 g of **2b** (6.5 mmol) is prepared with 75 mL of anhydrous THF. Following this, (1,3-dioxolan-2-ylmethyl)triphenylphosphonium bromide (6.05 g, 14 mmol) and 18-crown-6 (26 mg, 0.1 mmol) are added, the system is closed and the mixture is stirred for another 30 minutes. The third neck of the flask was opened under a positive flow of argon and NaH (60% dispersion in mineral oil, 2.5 g) was added in small portions over the course of 20 minutes. The flask was closed again and the mixture was stirred

for 3 hours at room temperature. The reaction was quenched with 4% water in THF until no more gas evolution was observed. The mixture was extracted with AcOEt (3×100 mL). The combined organic phase was dried over anhydrous sodium sulfate and the solvent was removed under vacuum, the residue was purified by silica gel chromatography (Toluene:EtOAc = 3:1) to give a yellow oily liquid consisting of (E)-3-(4-(bis(2-((tetrahydro-2H-pyran-2-yl)oxy)ethyl)amino)phenyl)acrylaldehyde (2c) (1.8g). LC-MS (ESI) m/z: Found 404.20; calculated for $[M+H]^+$: 404.24. Rt= 6.43 minutes.

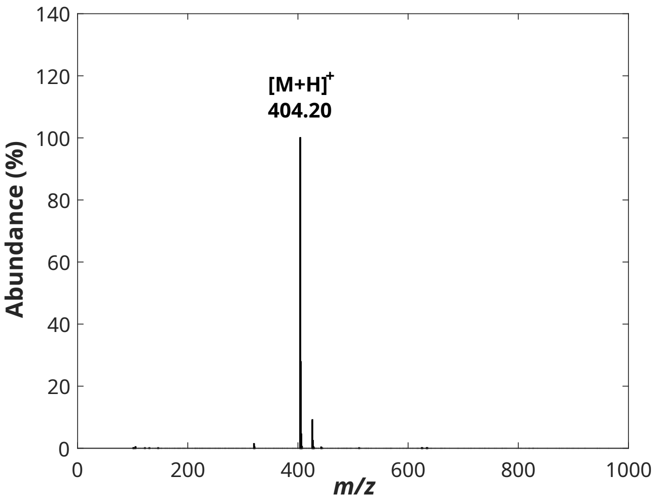

Figure S10: Mass spectrum of **2c**.

A round bottom flask is loaded with 720 mg of malononitrile (11 mmol) are added to a solution of 404 mg of **2c** (1 mmol) in EtOH (100 mL), no catalyst was needed for this Knoevenagel condensation. The mixture was stirred overnight at room temperature. The solvent was removed in a vacuum. The residue was purified by silica gel chromatography (Heptane:THF = 1:1) to give a red viscous oil consisting of **2d**. LC-MS (ESI) m/z: Found 451.20; calculated for $[M]^+$: 451.25. Rt= 3.33 minutes. The oil is dissolved in 2% HCl in EtOH and stirred at room temperature for 3 hours. The mixture is neutralized with K_2CO_3, filtered and concentrated under vacuum. The residue was purified by silica gel chromatography (diethyl ether:iPrOH = 9:1) to give a red viscous oil consisting of (E)-2-(3-(4-(bis(2-hydroxyethyl)amino)phenyl)allylidene)malononitrile (**2e**). LC-MS (ESI) m/z: Found 284.10; calculated for $[M+H]^+$: 284.14. Rt= 3.99 minutes. ^{1}H NMR (400 MHz, CD_3CN) δ: 7.72 (d, J = 11.8 Hz, 1H), 7.52 (d, J = 8.8 Hz, 2H), 7.32 (d, J = 14.8 Hz, 1H), 7.00 (ddd, J = 14.8, 11.9, 0.9 Hz), 6.79 (d, J = 8.7 Hz, 2H), 3.71 (d, J = 5.3 Hz, 4H), 3.59 (t, J = 5.7 Hz, 4H).

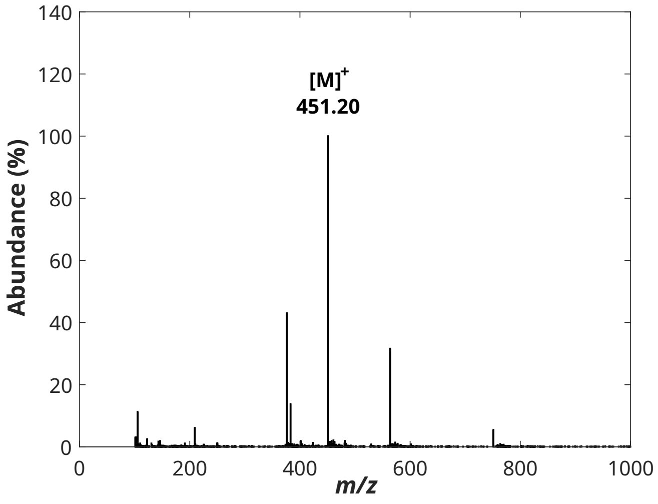

Figure S11: Mass spectrum of **2d**.

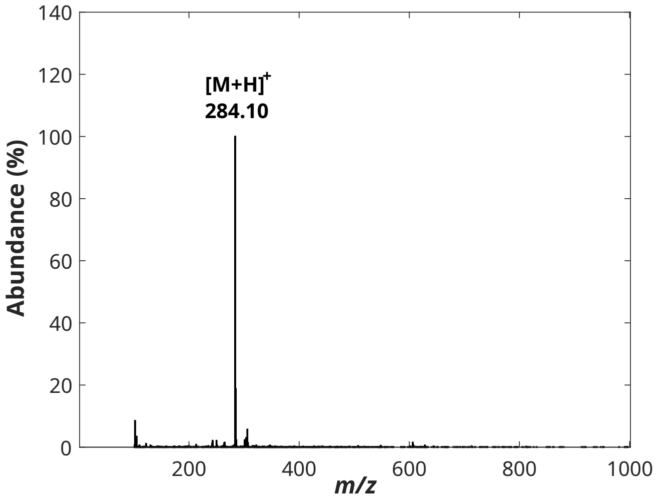

Figure S12: Mass spectrum of **2e**.

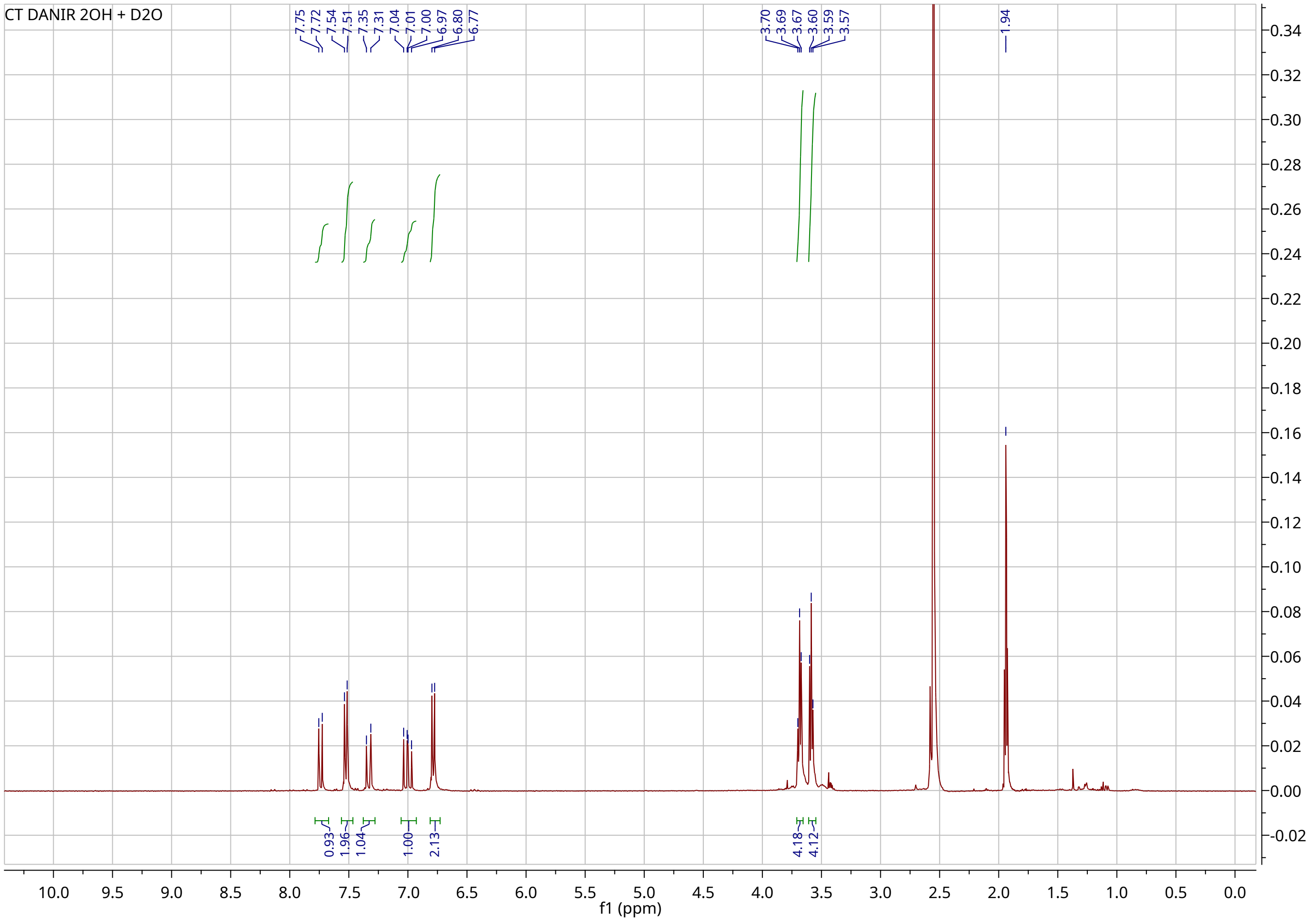

Figure S13: ^{1}H NMR spectra of DANIR-2b(2OH) in CD_3CN/D_2O 99:1.

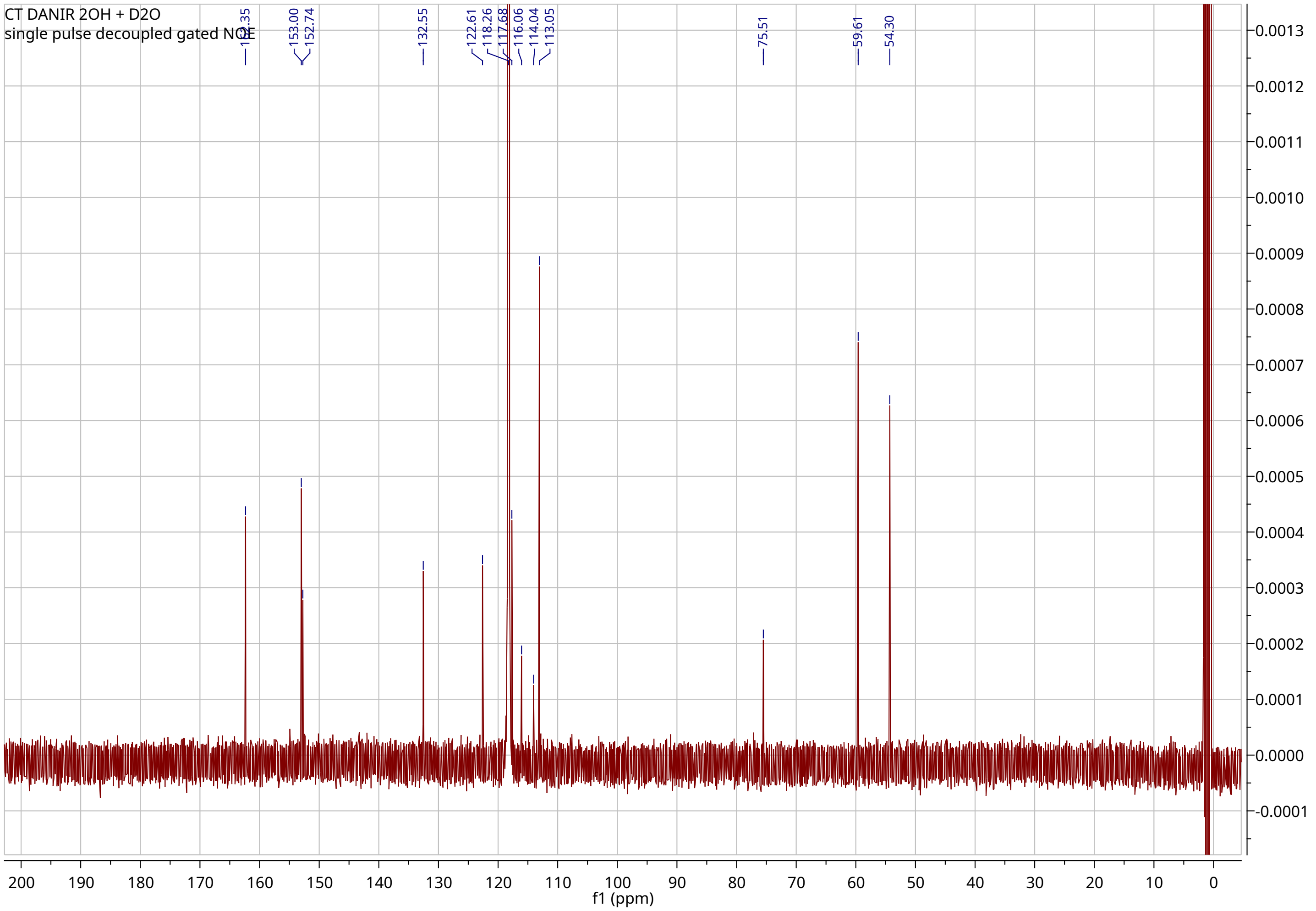

Figure S14: ^{13}C NMR spectra of DANIR-2b(2OH) in CD_3CN/D_2O 99:1.

3 Solubility measurements

The solubility of the two dyes was determined by measuring the absorption spectra of their saturated solutions. The concentration was then calculated based on the corresponding extinction coefficients with the Lambert-Beer law. In order to exclude contribution to the absorption spectra by undissolved dye, the solutions were filtered with 0.4 μm PTFE syringe filters (Avantor).

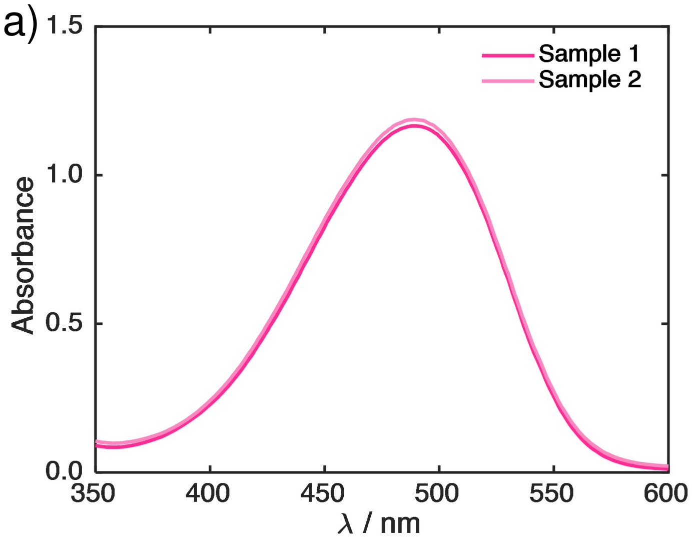

Figure S15: Absorption spectra of two samples containing (a) DANIR-2b(1OH) and (b) DANIR-2b(2OH) dissolved in water and measured in a 1 mm quartz cuvette. The latter was twice diluted to reduce the noise due to the high absorbance of the sample.

4 Extinction coefficients determination

The extinction coefficients of the two dyes was determined by measuring the absorption spectra of sample at known concentrations. For both dyes the measurement was performed on two replicates.

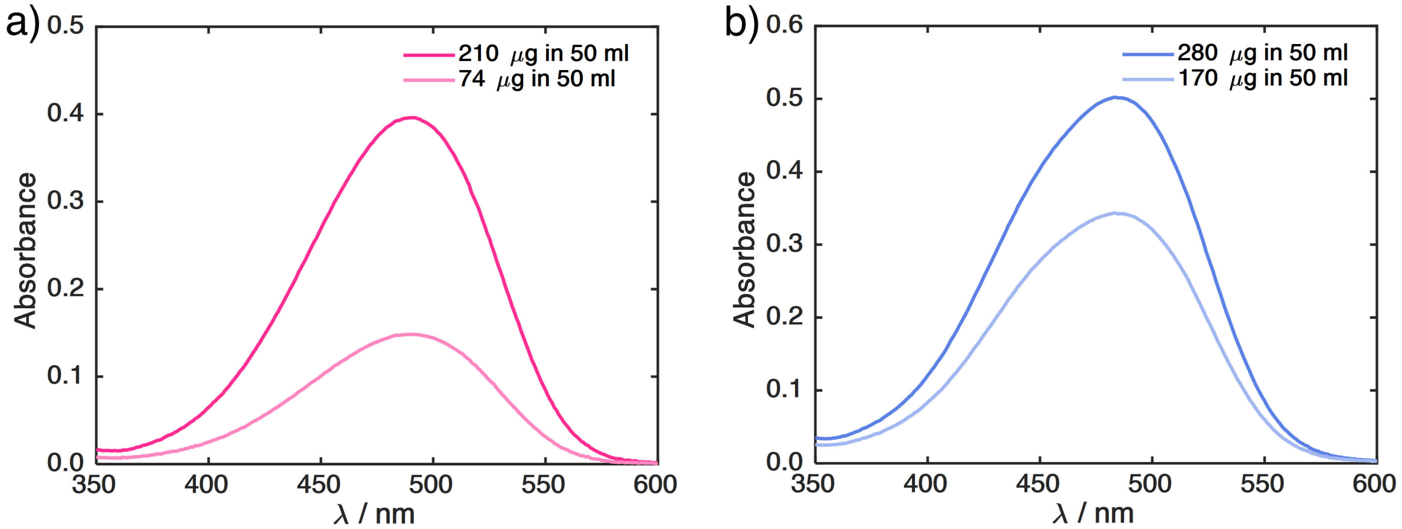

Figure S16: Absorption spectra of two samples containing (a) DANIR-2b(1OH) and (b) DANIR-2b(2OH) dissolved in water and measured in a 1 cm quartz cuvette.

5 Quantum yields determination

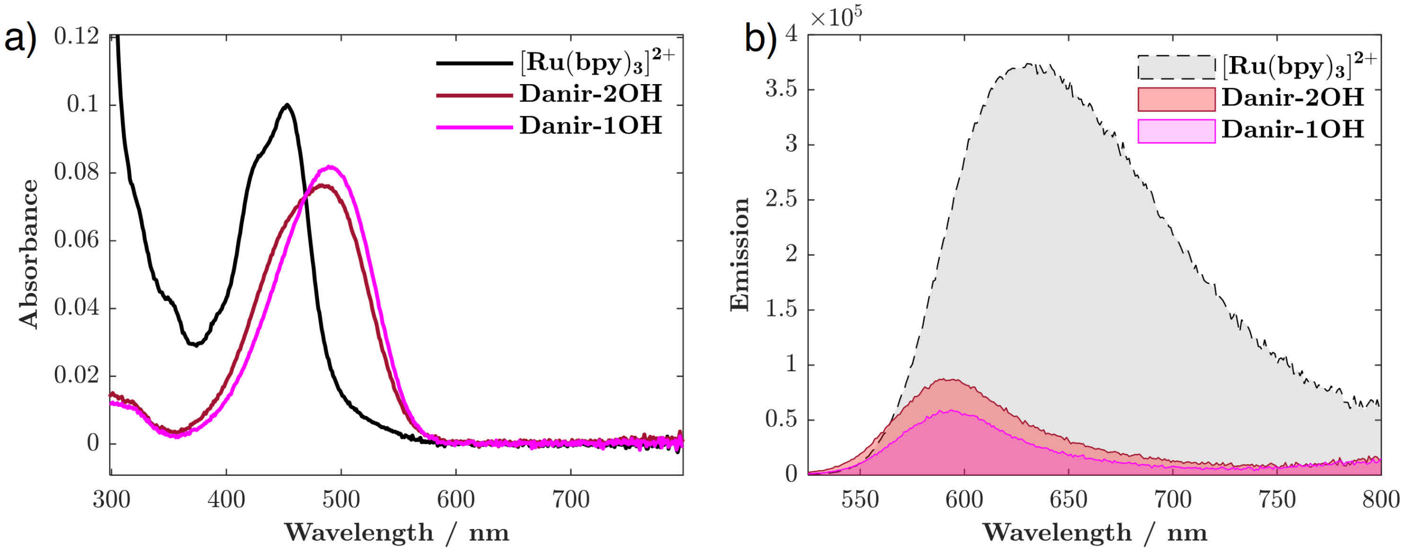

Figure S17: a) Absorption spectra of the samples of $[Ru(bpy)_3]^{2+}$, DANIR-2b(1OH) and DANIR-2b(2OH) in water used for the determination of the quantum yields. b) Emission spectra of of the samples of $[Ru(bpy)_3]^{2+}$, DANIR-2b(1OH) and DANIR-2b(2OH) in water excited at 470 nm.

6 Absorption and emission spectra

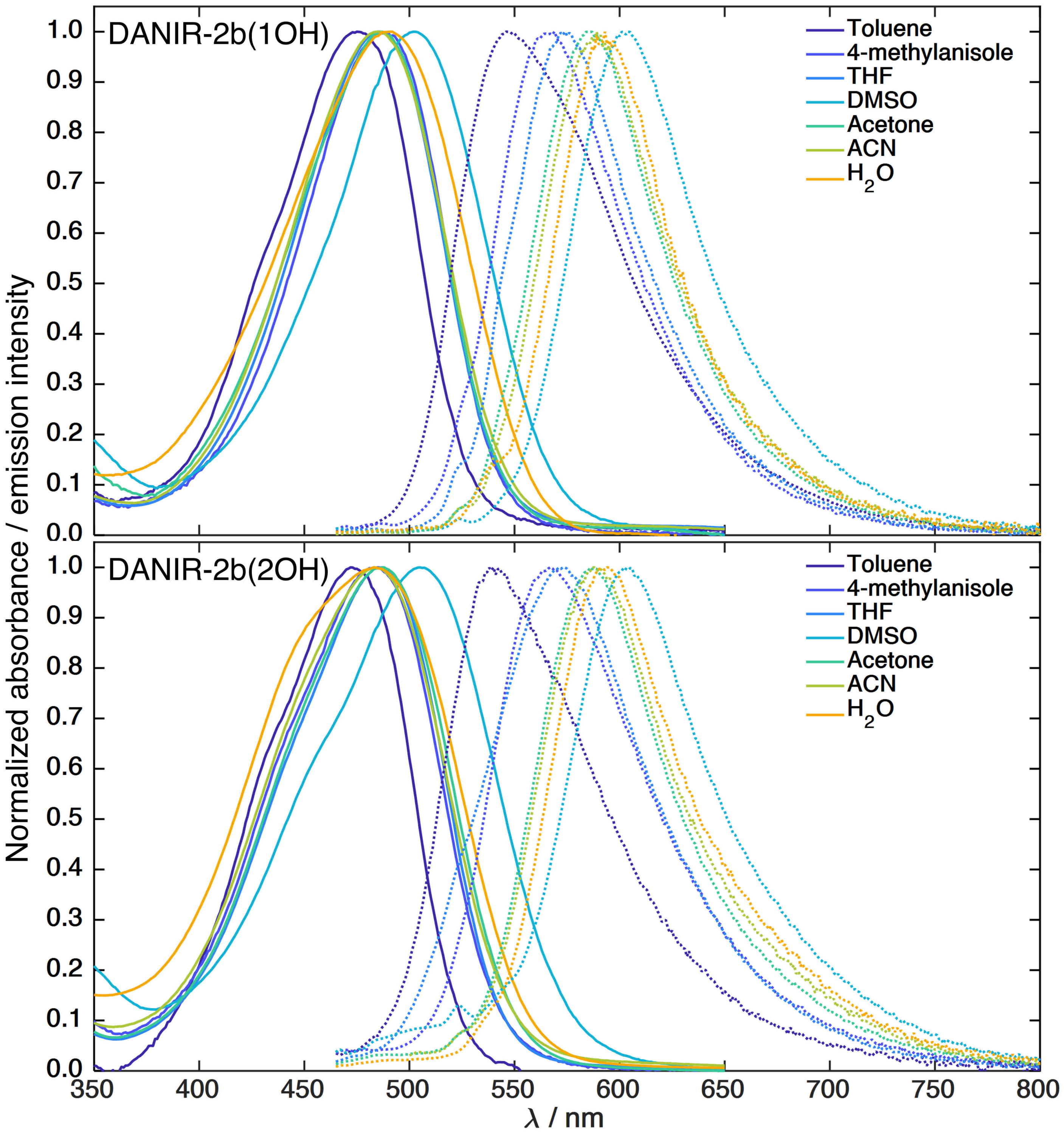

Figure S18: Normalized absorption (continuous lines) and emission (dashed lines) spectra of (top) DANIR-2b(1OH) and (bottom) DANIR-2b(2OH) in a series of solvents characterized by different polarities. THF: tetrahydrofurane, DMSO: dimethylsulfoxide, ACN: acetonitrile.

7 Atomic force microscopy

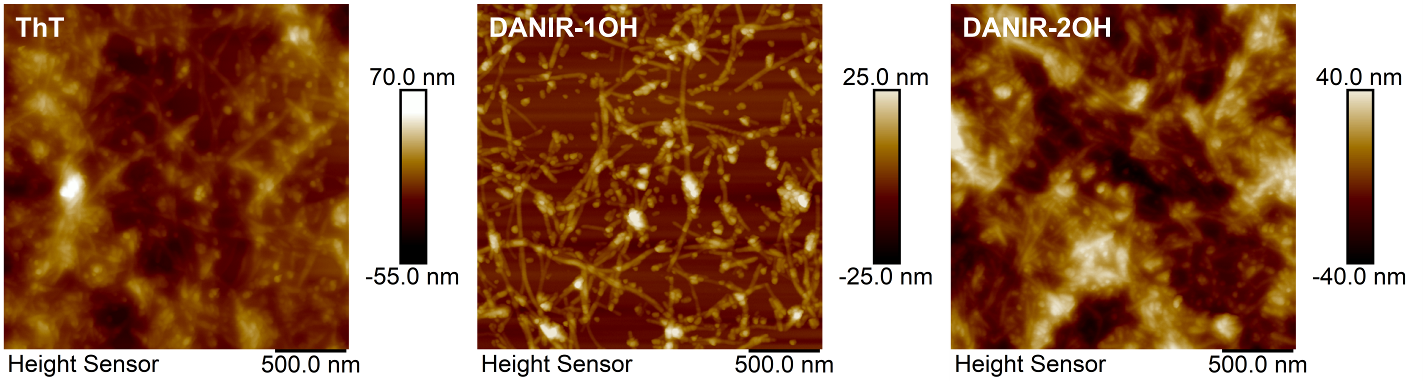

Figure S19: Representative atomic force microscopy images of hIAPP samples aggregated in presence of ThT, DANIR-2b(1OH) and DANIR-2b(2OH).

8 Photostability

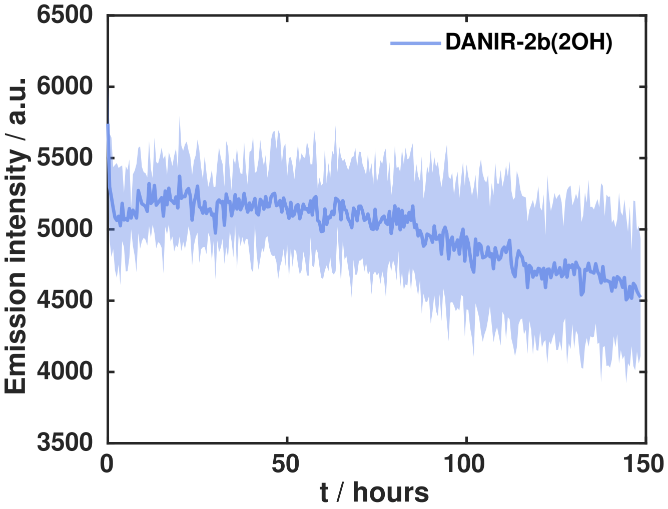

Figure S20: Emission intensity of DANIR-2b(2OH) in water recorded at 590 nm after excitation at 490 nm over 150 hours.

9 Titration experiments

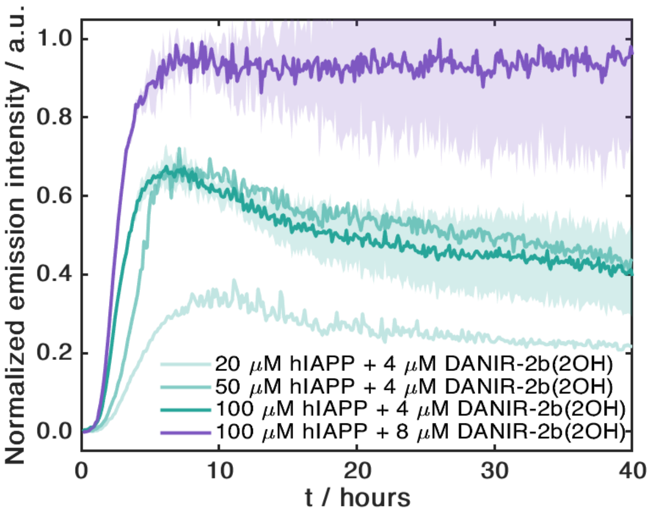

Figure S21: Aggregation kinetics of varying concentrations of hIAPP in 20 mM Tris (pH 7.4) monitored by 4 and 8 μM DANIR-2b(2OH). Data represent the mean of duplicate wells; shaded regions indicate standard deviation.

10 Time-Correlated Single Photon Counting

Given the low amount of sample and the low concentration of dyes, these experiments were performed with the emission monochromator slit width set at 20 nm to compensate for the little amount of light reaching the detector. Therefore, due to the low spectral resolution, it is not possible to unequivocally interpret the trends observed in the emission lifetimes. We instead decided to provide an average value across the entire emission spectrum, but we note that the variations in the emission lifetime of the dyes could point to the presence of different polymorphs or to the presence of multiple binding sites.

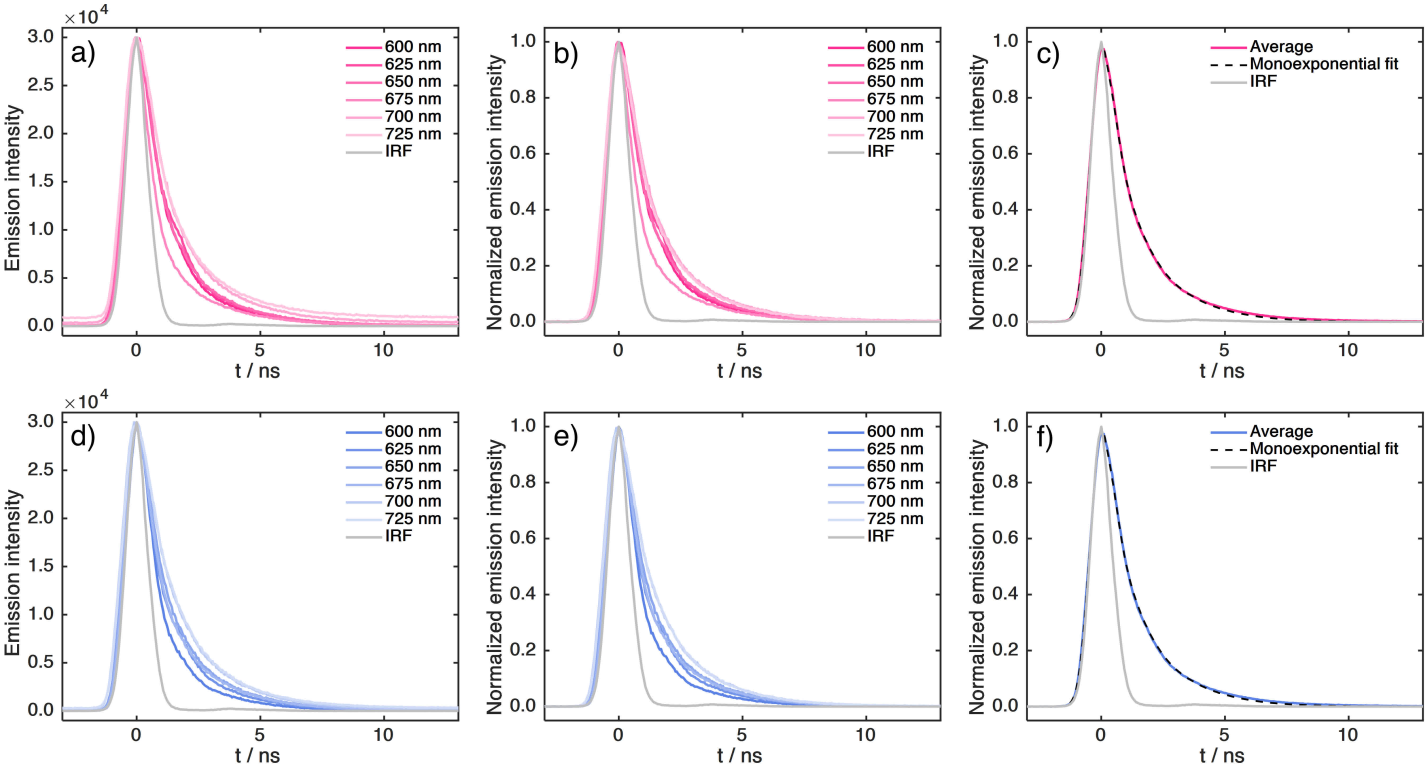

Figure S22: TCSPC traces of DANIR-2b(1OH) (a) and DANIR-2b(2OH) (d) bound to hIAPP fibrils for a series of different emission wavelength. Normalized traces of the two dyes (b,e). The averages of the six emission lifetimes of DANIR-2b(1OH) (c) and DANIR-2b(2OH) (f) overlaid with the monoexpoential fit. IRF: instrument response function.

11 Two-photon fluorescence microscopy

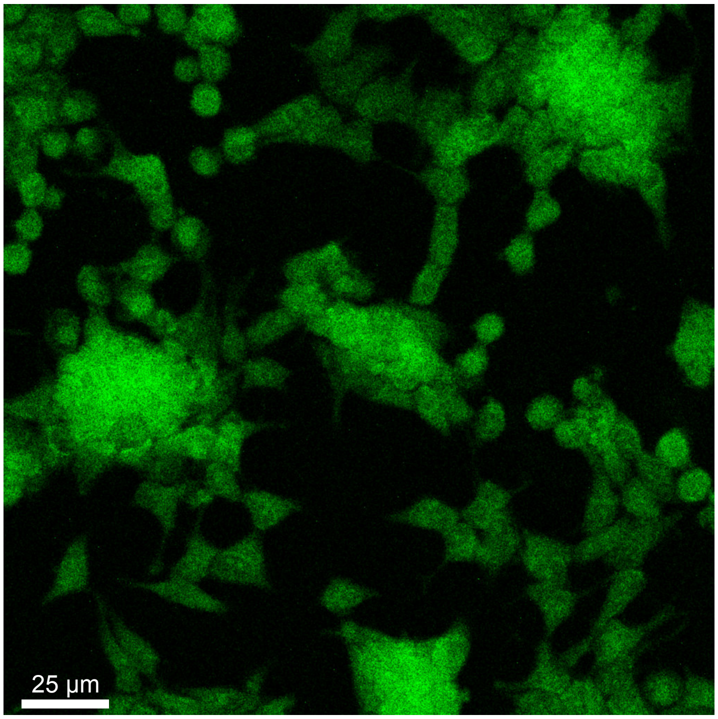

Figure S23: Two-photon fluorescence image of INS1-E cells stained with DANIR-2b(2OH). Fluorescence was measured using a two-photon excitation wavelength of 980 nm and emission was measured between 541 nm and 611 nm.

12 S/N determination

The S/N values for the kinetic traces of hIAPP and pIAPP tracked using DANIR-2b(2OH) and ThT were calculated by dividing the signal intensity at the plateau (or the maximum, in case of signal drop) by the signal intensity of the lag phase. In the case of the ThT data, the data points for the lag phase were chosen avoiding the instrument artifact at early timescales. The S/N for DANIR-2b(2OH) was determined to be 6.8 for hIAPP and 1.4 for pIAPP. The S/N for ThT was determined to be 1.2 for hIAPP and for 1.0 pIAPP.

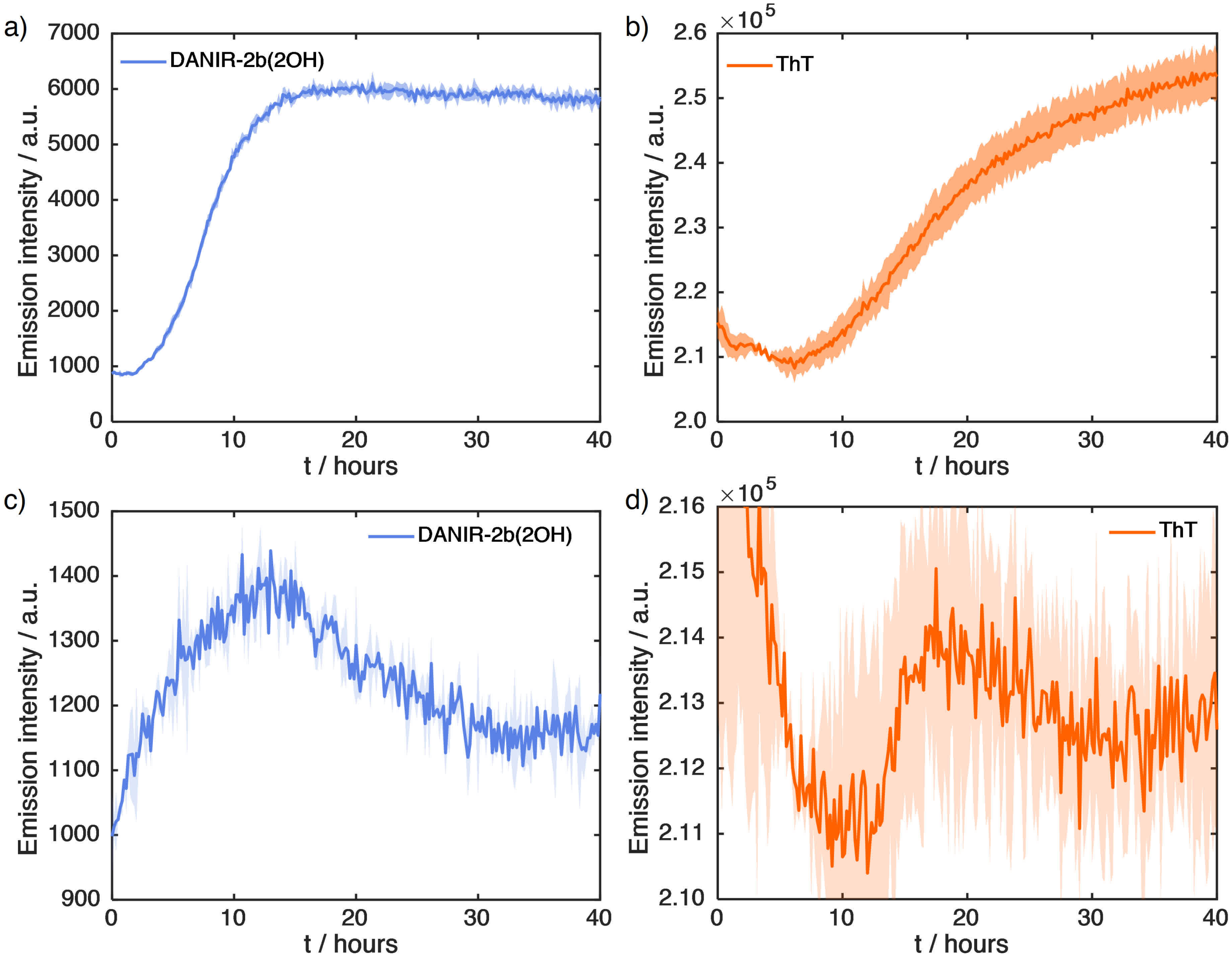

Figure S24: Aggregation kinetics of hIAPP tracked using (a) DANIR-2b(2OH) and (b)ThT. Aggregation kinetics of pIAPP tracked using (c) DANIR-2b(2OH) and (d)ThT.

References

(1) Watanabe, H.; Miki, Y.; Shimizu, Y.; Saji, H.; Ono, M. Synthesis and evaluation of novel two-photon fluorescence probes for in vivo imaging of amylin aggregates in the pancreas. *Dyes and Pigments* **2019**, *170*, 107615.
